# Metabolic model guided CRISPRi identifies a central role for phosphoglycerate mutase in *Chlamydia trachomatis* persistence

**DOI:** 10.1128/msystems.00717-24

**Published:** 2024-06-28

**Authors:** Niaz Bahar Chowdhury, Nick Pokorzynski, Elizabeth A. Rucks, Scot P. Ouellette, Rey A. Carabeo, Rajib Saha

**Affiliations:** 1 Chemical and Biomolecular Engineering, University of Nebraska-Lincoln, Lincoln, Nebraska, USA; 2 Department of Pathology, Microbiology, and Immunology, University of Nebraska Medical Center, Omaha, Nebraska, USA; LifeMine Therapeutics, Cambridge, Massachusetts, USA

**Keywords:** *C. trachomatis*, persistence, nutrient starvation, global stress response, metabolic bottleneck

## Abstract

Upon nutrient starvation, *Chlamydia trachomatis* serovar L2 (CTL) shifts from its normal growth to a non-replicating form, termed persistence. It is unclear if persistence reflects an adaptive response or a lack thereof. To understand this, transcriptomics data were collected for CTL grown under nutrient-replete and nutrient-starved conditions. Applying K-means clustering on transcriptomics data revealed a global transcriptomic rewiring of CTL under stress conditions in the absence of any canonical global stress regulator. This is consistent with previous data that suggested that CTL’s stress response is due to a lack of an adaptive response mechanism. To investigate the impact of this on CTL metabolism, we reconstructed a genome-scale metabolic model of CTL (iCTL278) and contextualized it with the collected transcriptomics data. Using the metabolic bottleneck analysis on contextualized iCTL278, we observed that phosphoglycerate mutase (*pgm*) regulates the entry of CTL to the persistence state. Our data indicate that *pgm* has the highest thermodynamics driving force and lowest enzymatic cost. Furthermore, CRISPRi-driven knockdown of *pgm* in the presence or absence of tryptophan revealed the importance of this gene in modulating persistence. Hence, this work, for the first time, introduces thermodynamics and enzyme cost as tools to gain a deeper understanding on CTL persistence.

**IMPORTANCE:**

This study uses a metabolic model to investigate factors that contribute to the persistence of *Chlamydia trachomatis* serovar L2 (CTL) under tryptophan and iron starvation conditions. As CTL lacks many canonical transcriptional regulators, the model was used to assess two prevailing hypotheses on persistence—that the chlamydial response to nutrient starvation represents a passive response due to the lack of regulators or that it is an active response by the bacterium. K-means clustering of stress-induced transcriptomics data revealed striking evidence in favor of the lack of adaptive (i.e., a passive) response. To find the metabolic signature of this, metabolic modeling pin-pointed pgm as a potential regulator of persistence. Thermodynamic driving force, enzyme cost, and CRISPRi knockdown of pgm supported this finding. Overall, this work introduces thermodynamic driving force and enzyme cost as a tool to understand chlamydial persistence, demonstrating how systems biology-guided CRISPRi can unravel complex bacterial phenomena.

## INTRODUCTION

*Chlamydia trachomatis* serovar L2 (CTL) is a Gram-negative obligate intracellular human pathogen (1) causing an estimated 1.7 million new genital tract infections annually (2). CTL replicates in a specialized membrane compartment, termed the inclusion, and uses different strategies to survive in the host intracellular environment (3). During its development cycle, CTL can alternate between the extracellular infectious elementary body (EB) and the intracellular non-infectious reticulate body (RB) (4). EBs enter epithelial cells and differentiate into RBs inside the inclusion. After several cycles of replication, RBs undergo an asynchronous secondary differentiation to create new EBs, which promote subsequent rounds of host cell infection (5).

When RBs experience nutrient starvation, they enter a reversible state of growth arrest, named persistence (6). RBs in the persistent state exhibit a distinct and enlarged morphological form, referred to as an aberrant reticulate body (ARB). ARBs remain metabolically active (7). Experimentally, chlamydial persistence can be induced with the addition of the cytokine interferon-γ (8), tryptophan starvation (9), iron starvation (10), or other stressors (11). Despite these studies, the fundamental nature of persistence is still puzzling. Whether persistence is an active or passive response is not clear. In evolving to obligate intracellular dependence, CTL has eliminated many genes from its chromosome, including canonical stress regulators like the stringent response and σ^S^ (12). One hypothesis is that CTL enters persistence as it lacks such canonical regulators (13–15). An extension of this hypothesis is that persistence is effectively a substitute for the lack of a conventional global response regulator. A recent study (16) compared iron and tryptophan starvation to nutrient-replete conditions. While there were significant transcriptomic differences between control and starvation conditions, it is still not clear if those changes were a directed and evolved response. To gain a holistic understanding about the mechanism of CTL persistence, it is pertinent to understand the metabolic landscape that occurs alongside the transcriptional changes. However, there is a dearth of experimental studies investigating the whole CTL metabolic landscape due to the challenging nature of working with an obligate intracellular pathogen. Thus, a systems biology approach may be useful to understand CTL metabolism during persistence.

Genome-scale metabolic models (GSMs) have been widely used in similar systems biology studies (17, 18). A GSM captures annotated metabolic reactions within a biological system and can predict reaction fluxes using flux balance analysis (FBA) (19), flux variability analysis (FVA) (20), and parsimonious FBA (pFBA) (21). To date, GSMs developed for different pathogens were successful in predicting metabolic adaptations of *Mycobacterium tuberculosis* (22), *Acinetobacter baumannii* (23), *Klebsiella pneumoniae* (24), and *Helicobacter pylori* (25). GSMs were also successful in predicting different virulence factors such as lipid A modifications of *Pseudomonas aeruginosa* (26) and substrate utilization patterns of *Staphylococcus aureus* (27). Moreover, GSMs successfully predicted novel drug targets for *Salmonella typhimurium* (28) and *Campylobacter jejuni* (29). Recently, a GSM was used to characterize the metabolic differences between chlamydial EBs or RBs under normal growth and development conditions and yielded the expected result that RBs are more metabolically active than EBs (30). We hypothesize that nutrient starvation will alter CTL metabolism in a manner that is distinct from the metabolic activities of EBs and RBs. Hence, we reconstructed the most comprehensive GSM of CTL, iCTL278.

To study the nature of persistence, persistence-specific “omics” data needed to be overlayed with the iCTL278. This process is called contextualization of the GSM. Without contextualization, GSM may predict unrealistic reaction fluxes (31), erroneous cellular phenotypes (32), or inaccurate growth rate patterns (33). Two different approaches are available for contextualization: switch [e.g., GIMME (34), iMAT (35), MADE (36), and RIPTiDe (37)] or valve [e.g., E-Flux (38), PROM (39), and EXTREAM (40)] approaches. While switch approaches display a binary nature, resulting in the active-or-inactive status of a reaction, valve approaches provide more flexibility by making the reaction flux proportional to the abundance of associated transcripts/proteins.

In this work, to perform systems-level investigations of CTL persistence, transcriptomics data were used for nutrient-sufficient and nutrient-starved conditions. To gain further insights from these transcriptomics data, K-means clustering algorithm was implemented, resulting in the identification of four core components of the CTL transcriptome. Furthermore, a statistical correlation study revealed a global transcriptomic rewiring of CTL under nutritional stress conditions. This, along with a lack of global stress regulators in CTL, revealed the lack of an adaptive response as the primary reason of CTL’s entry to persistence. To further understand how this global transcriptomic response impacts CTL metabolism, contextualized GSMs were reconstructed using the E-flux algorithm. Our recently developed metabolic bottleneck analysis (MBA) (40) identified phosphoglycerate mutase (*pgm*) as a candidate regulator of entry of CTL into persistence. The data revealed that *pgm* activity had the highest thermodynamics driving force and lowest enzymatic cost. To validate this finding, we used CRISPRi to block transcription of *pgm* and subsequently performed starvation experiments, which revealed enhanced sensitivity of *pgm* knockdown to starvation conditions. The outcome supported the prediction from MBA, thermodynamics driving force, and enzyme cost analysis. Additional systems-level investigation pinpointed the cellular impact of persistence, which is to prime itself for rapid growth upon the availability of nutrients. Overall, this metabolic model-guided study, for the first time, examined CTL persistence through the lens of thermodynamic driving force and enzymatic cost and will work as a blueprint to investigate phenotypical and genotypical changes associated with other microbial infections.

## RESULTS AND DISCUSSION

### *C. trachomatis* L2 genome-scale metabolic model development and analysis

**Fig 1:**
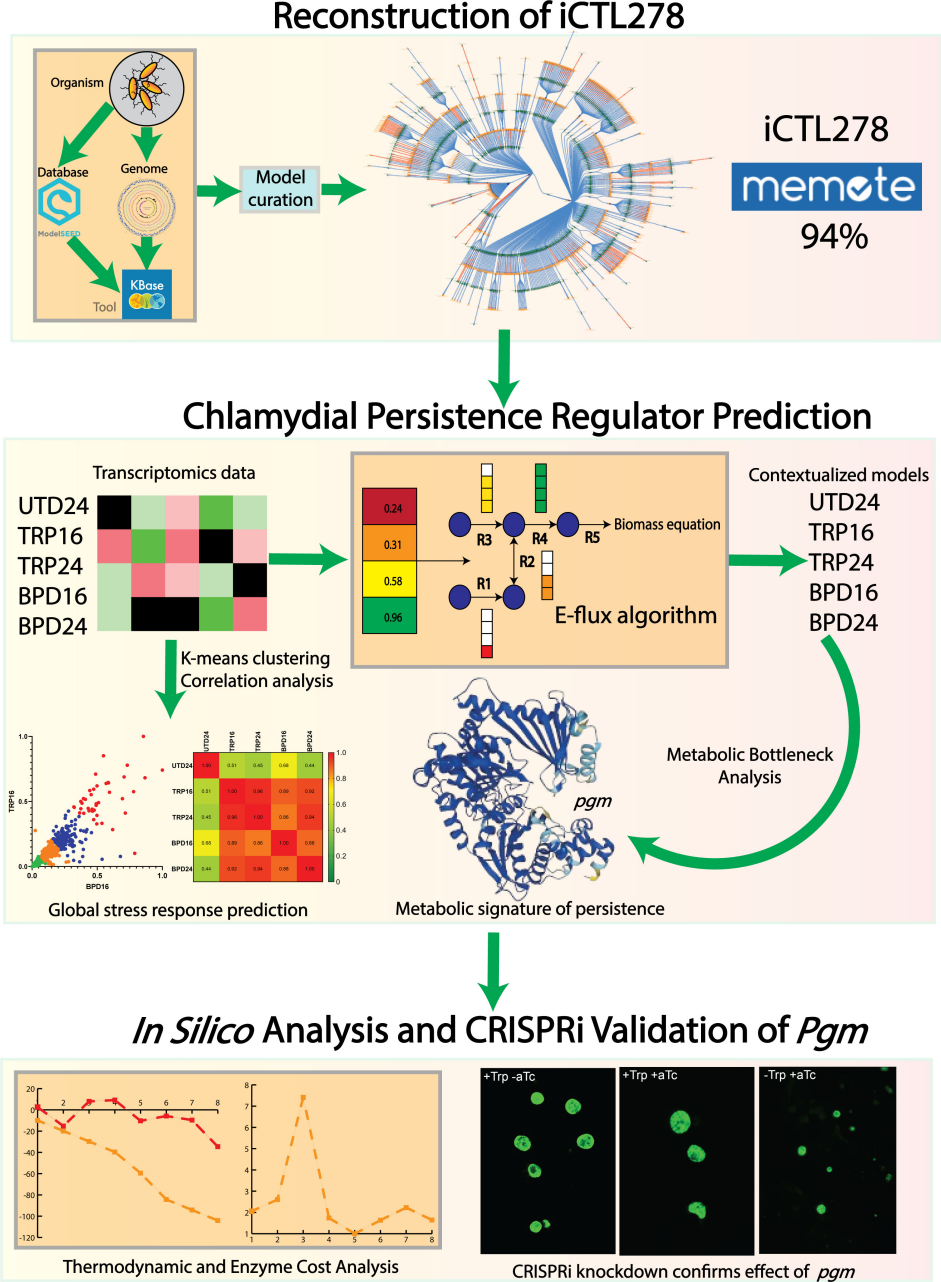
Overall process of the model reconstruction, refinement, and subsequent analysis/validation. Kbase, NCBI RefSeq, and ModelSEED database were used to reconstruct the initial model. After standard model curations, a high-quality GSM was ready to use. Later, condition-specific transcriptomics data were incorporated with GSM through E-flux algorithm, which resulted in five contextualized models. These models were used to find metabolic bottlenecks and persistence mechanism. K-mean clustering algorithm applied on the transcriptomics data revealed a global stress response mechanism. Thermodynamics and enzyme cost analysis, along with *in vitro* experimentation, reveal the regulatory role of *pgm* in CTL persistence.

**Fig 2:**
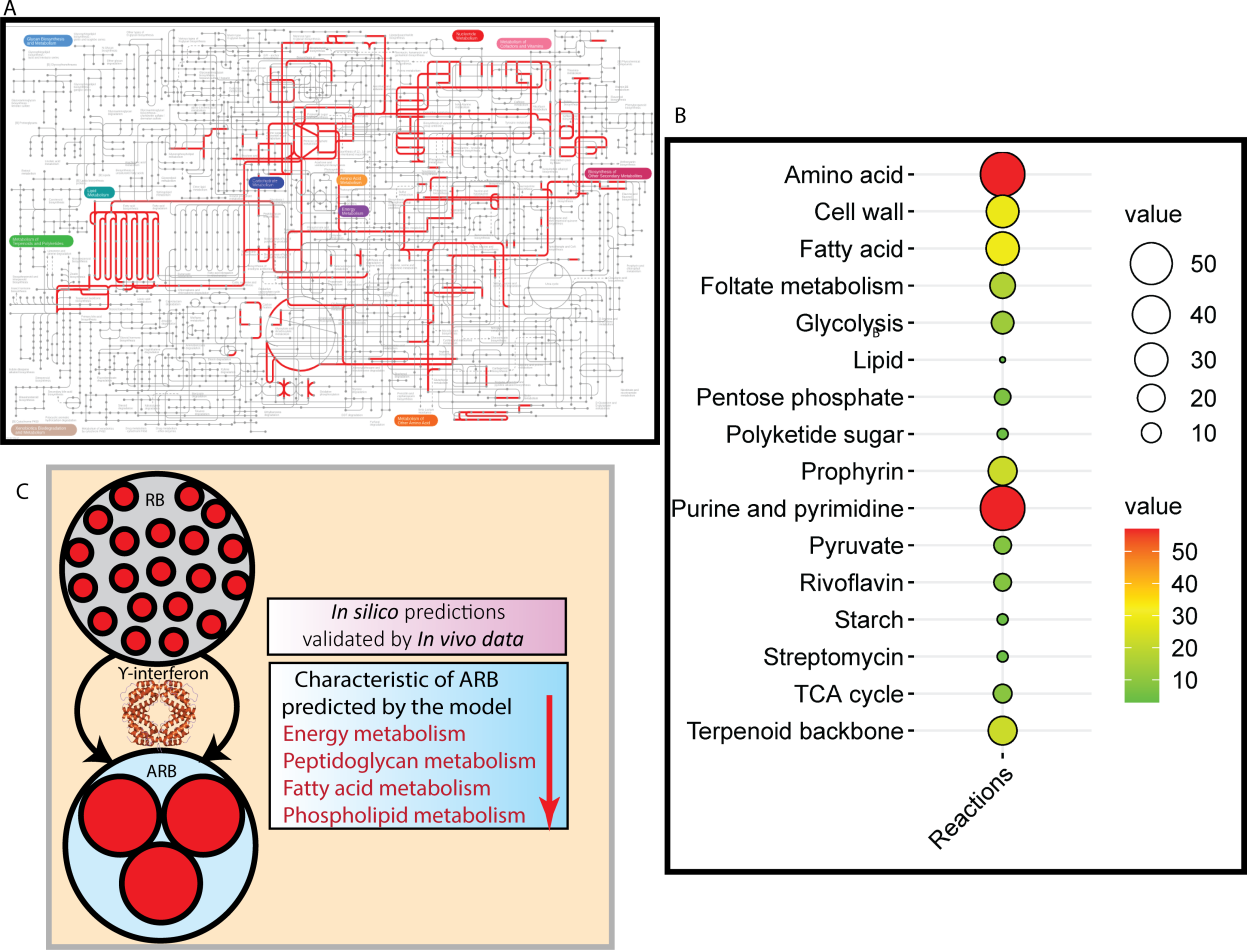
An overview of the metabolic network captured in the CTL genome-scale metabolic model and the result of model validation. (**A**) Visualization of the GSM metabolic network was performed using iPath3 (47). This metabolic network provides an idea regarding the limited metabolic capability of CTL. (**B**) A balloon plot representing a number of reactions in different pathways in the iCTL278. (**C**) Predictions for contextualized iCTL278 matched well with the experimental observations.

To analyze the metabolism of CTL in different stress conditions, we reconstructed a GSM of CTL using the NCBI RefSeq genome annotation (KBase Genome ID: GCF_000068585.1) from KBase (41). After reconstructing the draft GSM and adding reactions from the previously reconstructed model (30), a literature search was performed regarding the CTL-specific biomass composition. With information unavailable, a template Gram-negative biomass equation was used as the objective function of FBA. We performed a sensitivity analysis of each of the biomass constituents by increasing the coefficient by 10% as described in the literature (42) to assess its impact on the biomass growth rate. The analysis indicated only cobamide, peptidoglycan, and acyl carrier protein (ACP) impacted the biomass growth rate (Table S1). This analysis justifies the use of a template Gram-negative biomass equation, as the growth rate is not very sensitive to the stoichiometry of major biomass components such as amino acids, fatty acid, lipid, DNA, and RNA. This draft model did not capture some known metabolic functionality of CTL. For example, CTL has an incomplete tricarboxylic acid(TCA) cycle and must obtain malate from the host (43). However, the malate transport reaction was missing in the draft model. Similarly, CTL also obtains glucose 6-phosphate from the host cell (43). To account for these gaps in the metabolic network of CTL, we performed gap-filling using our previously developed tool OptFill (44) and added transport reactions for both malate and glucose 6-phosphate to the model. CTL partially depends on the host cell for ATP and NAD+ (43), and associated transport activities were added to the model to account for these biological needs. Overall, the curated model, iCTL278, consisted of 729 metabolites, 692 reactions, and 278 genes. We performed MEMOTE testing (45) for iCTL278, and it returned a score of 94%. The model is fully mass balanced and stoichiometrically consistent. The full MEMOTE report can be found in the Text S1. The MEMOTE score comparison between iCTL278 and the previously published CTL model is shown in Fig. S1A. In addition to performing MEMOTE, we also verified other known metabolic traits of CTL, such as the iCTL278 requirement of guanosine triphosphate (GTP), cytidine triphosphate (CTP), and uridine triphosphate (UTP) to sustain biomass growth (46). CTL is an auxotroph for most amino acids and can only biosynthesize alanine, aspartate, and glutamine using dicarboxylates from the TCA cycle (43). The rest of the amino acids needs to be acquired from the host cell. iCTL278 recapitulated these metabolic traits for amino acids. Overall, iCTL278 captured all the known metabolic characteristics of CTL. The model reconstruction process is shown in Fig. 1, the metabolic networks of the model are shown in Fig. 2A, and the number of reactions in different pathways is shown in Fig. 2B.

### Model validation using experimental proteomics data

Although the MEMOTE testing of iCTL278 returned a score of 94%, it was still necessary to determine if iCTL278 could capture the known phenotypes of CTL upon contextualization. In a previous study (38), a valve-based approach using the E-flux algorithm, which connects reaction activities linearly with the gene expression levels, accurately predicted different cellular phenotypes regarding fatty acid biosynthesis of *M. tuberculosis*, a well-known pathogenic bacterium. Therefore, a previously published study on quantitative normalized protein profiling of interferon-γ-treated CTL (8), despite its limited effort to confirm the induction of aberrance, was used to reconstruct two contextualized models using the E-flux algorithm (38), RB, and ARB models. The latter was under stress induced by interferon-γ. The proteomics data set covered all 278 proteins of the iCTL278, thus ensuring a high degree of coverage of proteomics data for the model.

When flux distribution was calculated for both RB and ARB, similar to previous studies, flux through energy metabolism was reduced in the ARB compared to RB (8). As CTL enters persistence, cell division is arrested, and metabolism is reduced to a minimum level such that only essential cellular functions are maintained. As GSMs are unable to directly calculate the concentration of different metabolites involved in a biochemical network, we used the flux-sum analysis (FSA) approach to predict the metabolic pool size of ATP in both RB and ARB for further verifications and found a lower ATP pool size in the ARB condition. The details of flux-sum analysis can be found in our previous work (33) and also in the Materials and Methods section. Thus, the reduced energy metabolism in ARB compared to the RB, a distinguishing feature of the persistence state, was successfully captured by the iCTL278.

As a further verification, we explored the peptidoglycan biosynthesis pathway of CTL. Peptidoglycan is essential for cell division and is evident only at the division septum in CTL but, unusually, is not a component of the cell wall (48). During stress conditions, CTL arrests its cell division (6). As cell division is arrested in the ARB, the model predicted reduced peptidoglycan biosynthesis in ARB compared to RB, which is consistent with a previous study showing reduced expression of cell division components under tryptophan starvation (49). CTL relies on both *de novo* biosynthesis and salvage from the host for fatty acid and phospholipid to produce its membrane phospholipids, which are also crucial for cell division (50). When fatty acid biosynthesis reactions in both RBs and ARBs were compared, we found reduced activity in the fatty acid biosynthesis in ARBs compared to RBs, which reiterates what has previously been reported (51). This was expected given the predicted reduced requirement for fatty acid biosynthesis in the absence of bacterial replication.

Overall, iCTL278 recapitulates known phenotypes of ARBs and RBs upon contextualization. Therefore, iCTL278 is well suited to analyze the metabolism of CTL under different stress conditions. The summary of the validation result is shown in Fig. 2C.

### Effects of nutrient starvation on CTL growth and development

Our understanding of the molecular underpinnings behind the persistence of CTL is still limited. Despite employing numerous experimental methods to induce persistence, the result consistently shows similar ARB morphology, as well as disruptions in the transcriptome and proteome. Thus, specific alterations to transcriptional and translational profiles may precede, and thus induce, aberrant growth.

**Fig 3:**
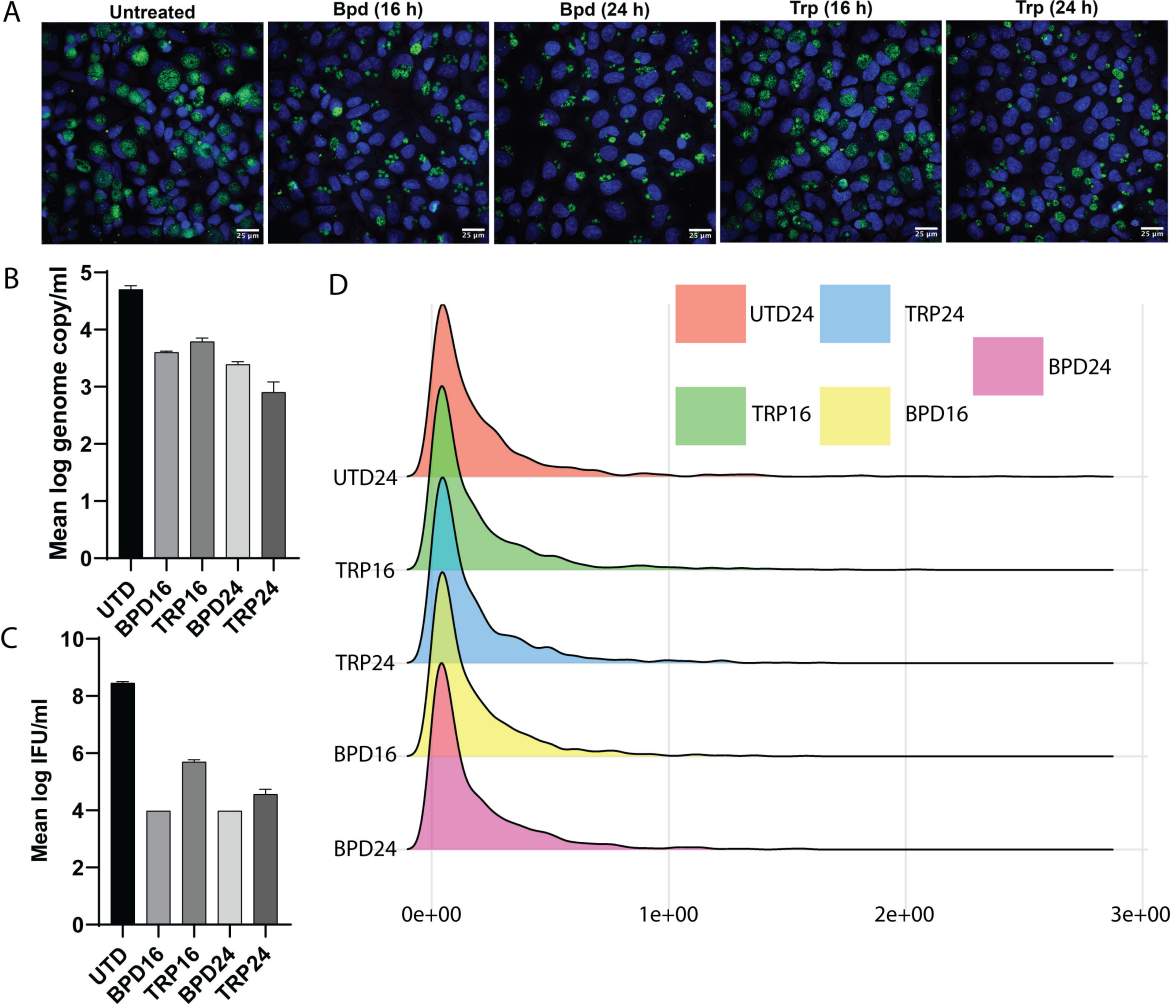
Morphology, genome-copy number, and infectious progeny data for different stage of CTL tryptophan and iron starvation. (A) CTL exhibits duration-dependent sensitivity to iron limitation and tryptophan (trp) starvation. Infected cells were starved for iron by treatment with the chelator 2,2-bipyridyl (Bpd) starting at either the time of infection or at 8 hours post-infection. Inclusions as indicated by staining for the chlamydial major outer membrane protein (MOMP) were allowed to develop for a total of 24 hours. Tryptophan starvation was started at the same time points as above, with indicated durations. Note the more significant delay in inclusion development by Chlamydia starved for 24 hours. (**B**) Genome copy number data of *euo* gene for UTD24, BPD16, TRP16, BPD24, and TRP24. (**C**) Infectious progeny data for UTD24, BPD16, TRP16, BPD24, and TRP24. (D) Distribution of gene expression values.

We assayed several distinguishing features of CTL persistence across the different treatment conditions, i.e., untreated (UTD24), 16 hours of iron chelation with the chelator 2,2-bipyridyl (Bpd; BPD16) or tryptophan starvation (TRP16), and 24 hours of iron (BPD24) or tryptophan starvation (TRP24). All treatments were performed over a 24-hour period. In the case of TRP16/BPD16, infection was induced after 8 hours, while for TRP24/BPD24, it was induced immediately at the start. One of those was the morphology of CTL, which was monitored by immunofluorescent confocal microscopy. Here, we confirmed that, in contrast to the untreated CTL inclusions at 24 hours post-infection (hpi; UTD24), all starvation strategies yielded smaller inclusions (Fig. 3A), which was associated with lower yields of inclusion-forming units, indicating severely delayed development that compromised the generation of infectious particles. We observed that BPD treatments resulted in aberrantly enlarged organisms. TRP inclusions were smaller, indicating a growth defect. We also analyzed genome copy number across all stress conditions. We observed significantly reduced genome equivalents (GEs) for all stress conditions compared to UTD24. Unsurprisingly, genome copy numbers of the chlamydial late-stage transcriptional regulator, *euo*, between BPD and TRP were similar (Fig. 3B). With regard to generation of infectious progeny, we observed a much more dramatic effect of BPD treatment than TRP starvation when stress was administered at *t* = 0 or 8 hours post-infection and maintained for 16 or 24 hours, respectively, indicating that iron starvation was more effective in inducing CTL developmental arrest relative to tryptophan depletion (Fig. 3C).

**Fig 4:**
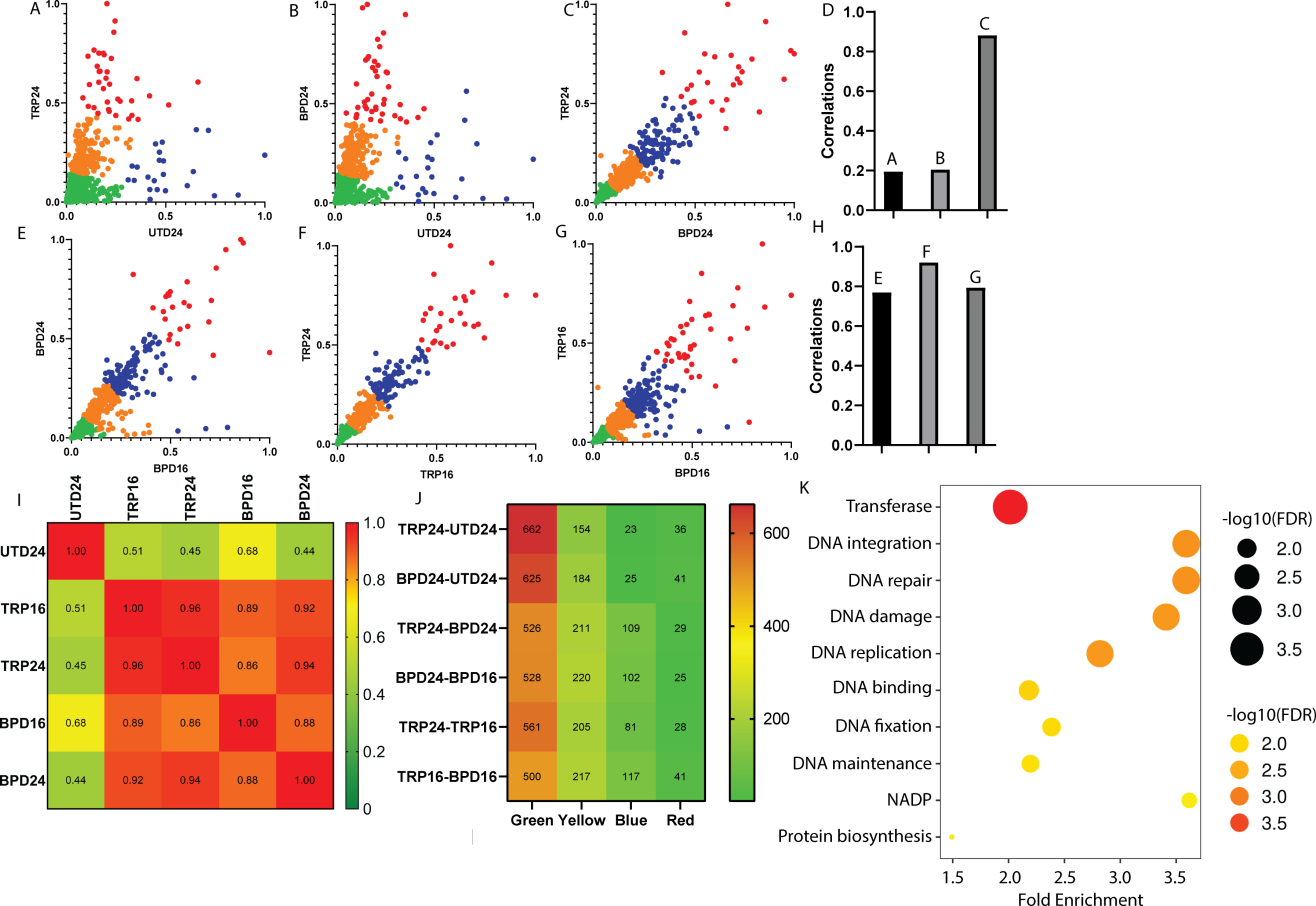
Pairwise K-mean clustering plots for different stress conditions. (**A**) Scatter plot of 24 hours of tryptophan starved (TRP24) and untreated (UTD24) CTL. (**B**) Scatter plot of 24 hours of iron starved (BPD24) and untreated (UTD24) CT. (**C**) Scatter plot of 24 hours of tryptophan starved (TRP24) and 24 hours of iron starved (BPD24). (**D**) Correlation of different scatterplots. These scatterplots and correlations between different stress conditions reveal that CTL may behave similarly under different stress conditions. (**E**) Scatter plot of 24 hours of iron starved (BPD24) and 16 hours of iron starved (BPD16) CTL. (**F**) Scatter plot of 24 hours of tryptophan starved (TRP24) and 16 hours of tryptophan starved (TRP16) CT. (**G**) Scatter plot of 16 hours of tryptophan starved (TRP16) and 16 hours of iron starved (BPD16). (**H**) Correlation of different scatterplots. These scatterplots and correlations between different stress conditions reveal that CTL may have a global transcriptomics response. (**I**) Pearson correlation matrix among different conditions. The correlation was calculated using two-tail test with 95% CI. (**J**) Heat map for a number of reactions in each cluster for different conditions. (**K**) Gene ontology enrichment chart for common genes in green cluster across all conditions.

To further analyze the impact of environmental stress on CTL, RNA sequencing was performed for each of the stress conditions, including an untreated control collected at 24 hpi (16). Many genes fall in the low expression level, and gene expression distribution also skewed heavily toward the low expression range (Fig. 3D). Subsequently, a pair-wise comparison of profiles was conducted to determine the level of similarities of transcriptomes across different stress conditions. Using scatter plots, we were able to visualize correlations between pairs of transcriptomics data. From comparisons of TRP24-UTD24 (Fig. 4A) and BPD24-UTD24 (Fig. 4B), we found a weak correlation for each case (Fig. 4D). However, TRP24-BPD24 (Fig. 4C) showed a strong correlation (Fig. 4D). This pointed to CTL may having a common global transcriptomic response to different stress conditions. To reveal more about the pattern of global transcriptomic response, we implemented an unsupervised machine-learning technique, K-means clustering, on TRP24-UTD24, BPD24-UTD24, and TRP24-BPD24 samples. K-means clustering identified four distinct clusters for each plot. Details of each cluster can be accessed in the Table S2.

As K-means clustering is an unsupervised learning algorithm, it may be difficult to decide if these clusters are biologically different or not. However, with the help of bioinformatics tools, we can decide the arbitrariness of each cluster. For that purpose, we performed gene set enrichment analysis, a well-established method to identify the uniqueness of clusters obtained from the K-means algorithm (52).

Genes were clustered based on function, with clusters in green indicating metabolism-related genes; yellow clusters consisting of cell signaling-related genes, and red and blue clusters indicating transcription- and translation-related genes, respectively. From Fig. 4A and C, it is evident that most of the genes in green clusters remained similarly expressed. However, genes in the yellow and red cluster were moderately or highly upregulated in the TRP24/BPD24 compared to the UTD24 (Fig. 4A and B). In contrast, genes in the blue clusters were upregulated in the UTD24 compared to the TRP24/BPD24 (Fig. 4A and B).

As green clusters are tightly clustered across all the conditions (Fig. 4A and C), these can be called “core” components, as identified previously (16). In contrast, blue, yellow, and red clusters were upregulated/downregulated across given conditions (Fig. 4A and C) and can be labeled as stress-specific “accessory” components (16). Some of the known tryptophan utilization genes such as *trpR*, *trpC*, and *trpS* fell in the yellow clusters and were thus moderately upregulated in TRP24 (Fig. 4A). Several studies predicted similar upregulation of tryptophan utilization genes under tryptophan starvation (15). *In vivo*, the amount of tryptophan available is often depleted by the pro-inflammatory cytokine interferon-γ through the transcriptional upregulation by the *ido1* gene, encoding the enzyme Indoleamine 2,3‐dioxygenase (IDO) that catabolizes tryptophan to kynurenine, which cannot be used by CTL. However, the microbiome in the relatively hospitable niche of the lower genital tract may provide tryptophan or more likely indole, which CTL can use for tryptophan biosynthesis via the salvage pathway (53). Indeed, several studies have shown that under tryptophan starvation, CTL can use indole supplemented in the media for conversion to tryptophan (53, 54).

Several stress-related genes, such as *ahpC* and *euo*, were also upregulated in the TRP24 compared to the UTD24 (Fig. 4A). Between these two genes, *ahpC* was significantly upregulated and fell in the red cluster, while *euo* was moderately upregulated and included in the yellow cluster. *AhpC* is a thiol-specific antioxidant peroxidase gene and is predicted to regulate redox homeostasis in CTL. A similar upregulation of *ahpC* is also observed in CTL exposed to interferon-γ stress (55). For *Chlamydia pneumoniae*, it was reported that the upregulation of *ahpC* would protect the bacteria against cytokine-induced reactive nitrogen intermediates, thus allowing *C. pneumoniae* to cause long-term infection (56). For *euo*, encoding a DNA-binding protein, a moderate upregulation was observed in CTL under interferon-γ induced stress. Euo is a predicted negative regulator of CTL genes involved in RB-to-EB differentiation (57). *IhfA* is a DNA-binding protein that alters DNA topology and was downregulated in the TRP24, thus, possibly contributing to other transcriptional effects in TRP24-treated samples (Fig. 4A).

In persistence, glycolysis mostly supports the increased production of starch and carbohydrate, with CTL mostly depending on the host cell for energy (43). As a result, reduced expression of TCA cycle genes, such as *sucABCD*, was expected and fell in the bottom left portion of the green cluster. A similar overall result was observed for BPD24-UTD24 (Fig. 4B). In the TRP24-BPD24 (Fig. 4C) scatterplot, the key difference is that the stress-related accessory genes were expressed more in line with the core genes, thus establishing a strong relation between two different stress conditions. Overall, the K-means clustering predicted clusters whose functionality matched closely with the literature. Thus, this clustering analysis will further serve as a “genome-wide library” to identify core and stress-related genes of CTL.

To gather more insights into the presence of a global stress response in CTL, we plotted other stress conditions, such as BPD24-BPD16 (Fig. 4E), TRP24-TRP16 (Fig. 4F), and TRP16-BPD16 (Fig. 4G). Interestingly, we noticed a very strong correlation in all the cases (Fig. 4H), supporting the proposed existence of global stress response of CTL. A Pearson correlation heat map (Fig. 4I) of normalized read counts of all genes among different conditions also indicated strong correlation between all the stress conditions. The number of reactions in each cluster for different conditions is shown in Fig. 4J. Common genes in green, yellow, blue, and red clusters were identified using Venn diagrams (Fig. S1B through E respectively). Gene ontology enrichment analysis for the common genes across green clusters is shown in Fig. 4K. The same analysis for the yellow cluster (Fig. S2A) and the red clusters (Fig. S2B) is provided in the supplementary information. Since the blue cluster has only one common gene (*ctl0256*), we could not perform the gene ontology enrichment analysis.

Notably, the strong correlation between transcriptomes associated with two distinct stresses is not unique to CTL. Analysis of publicly available transcriptomes from differently stressed *M. tuberculosis* and *Escherichia coli* revealed a similar correlation. We collected the transcriptional profile of *M. tuberculosis* exposed to *in vitro* lysosomal stress for 24 hours and 48 hours (58). From the scatterplot (Fig. S3A), we noticed a very high correlation (Fig. S3D). We also collected the transcriptional profile of *M. tuberculosis* exposed in zinc-limited medium and zinc replete medium (59). Similar to the previous case, the scatterplot (Fig. S3B) indicated a very high correlation (Fig. S3D). However, when we scatterplot (Fig. S3C) *M. tuberculosis* transcription data for lysosomal stress of 24 hours and zinc-limited medium, it showed a very low correlation (Fig. S3D). Thus, *M. tuberculosis* response to divergent stresses is customized to each stress. However, the transcriptional response for a single type of stress in *M. tuberculosis* progresses as a function of severity (i.e., duration) of the stress.

Unlike *M. tuberculosis*, *E. coli* demonstrated a single global stress response mechanism. Transcriptomics profile of 50 minutes of cold stress against 90 minutes of cold stress (Fig. S4A) for *E. coli* from the literature (60) showed a very strong correlation (Fig. S4D). Similarly, transcriptomics profile of 40 minutes of oxidative stress against 90 minutes of oxidative stress (Fig. S4B) for *E. coli* from the literature (60) also showed a very strong correlation (Fig. S4D). Thus, like *M. tuberculosis*, for temporal progression of similar stress, *E. coli* may have a similar stress response mechanism. Interestingly, the transcriptomics profile of 90 minutes of cold stress against 90 minutes of oxidative stress (Fig. S4C) showed a very strong correlation (Fig. S4D). Thus, under different stress responses, the transcriptional response of *E. coli* is generally conserved, similar to our data for CTL.

For *E. coli*, ppGpp is a global stress regulator (61). When an uncharged tRNA binds in the ribosome, the ribosome-associated RelA protein is activated to synthesize ppGpp (62), which acts as a global regulator of transcription by modulating transcription complexes at promoters (61). The stringent response serves to stop the synthesis of stable RNA species, such as rRNA and tRNA, to increase protein degradation pathways to maintain growth rate (62). Collectively, these responses serve to overcome the starvation. SpoT is a cytosolic bifunctional enzyme with ppGpp synthase and hydrolase activity that helps control the levels of ppGpp. CTL does not have homologs of *relA* and *spoT* and does not synthesize ppGpp (12). The loss of these genes has likely occurred through reductive evolution as a means for adapting to obligate intracellular environment. Thus, gene expression showing a strong correlation under various stress conditions, lack of homologs for *relA* and *spoT*, missing metabolic pathways to synthesize ppGpp, and elevated expression of stable RNA (13) indicate that CTL does not engage in a stringent response during starvation, manifesting as an overlapping transcriptional response during iron and tryptophan starvation.

### Impact of global stress response on *C. trachomatis* metabolism

We next sought to determine if this lack of stringent action translated to novel insights into the metabolic landscape of tryptophan- or iron-starved CTL. To answer this, we contextualized iCTL278 for UTD24, TRP16, TRP24, BPD16, and BPD24 conditions using the E-flux algorithm.

To confirm that the results from contextualized models are not the artifact of transcriptomics data, we calculated the correlation matrix between transcriptomics data predicted flux (detailed descriptions of experimental design are found in the “E-flux algorithm” sub-section of the Methods and Materials) and model-predicted flux and found very weak correlation between them (Fig. S5A). We also found that model-predicted flux distribution for different stress conditions is highly correlated (Fig. S5B). This supports the previously observed high correlation among different stress conditions from the transcriptomics data analysis(Fig. S4I).

**Fig 5:**
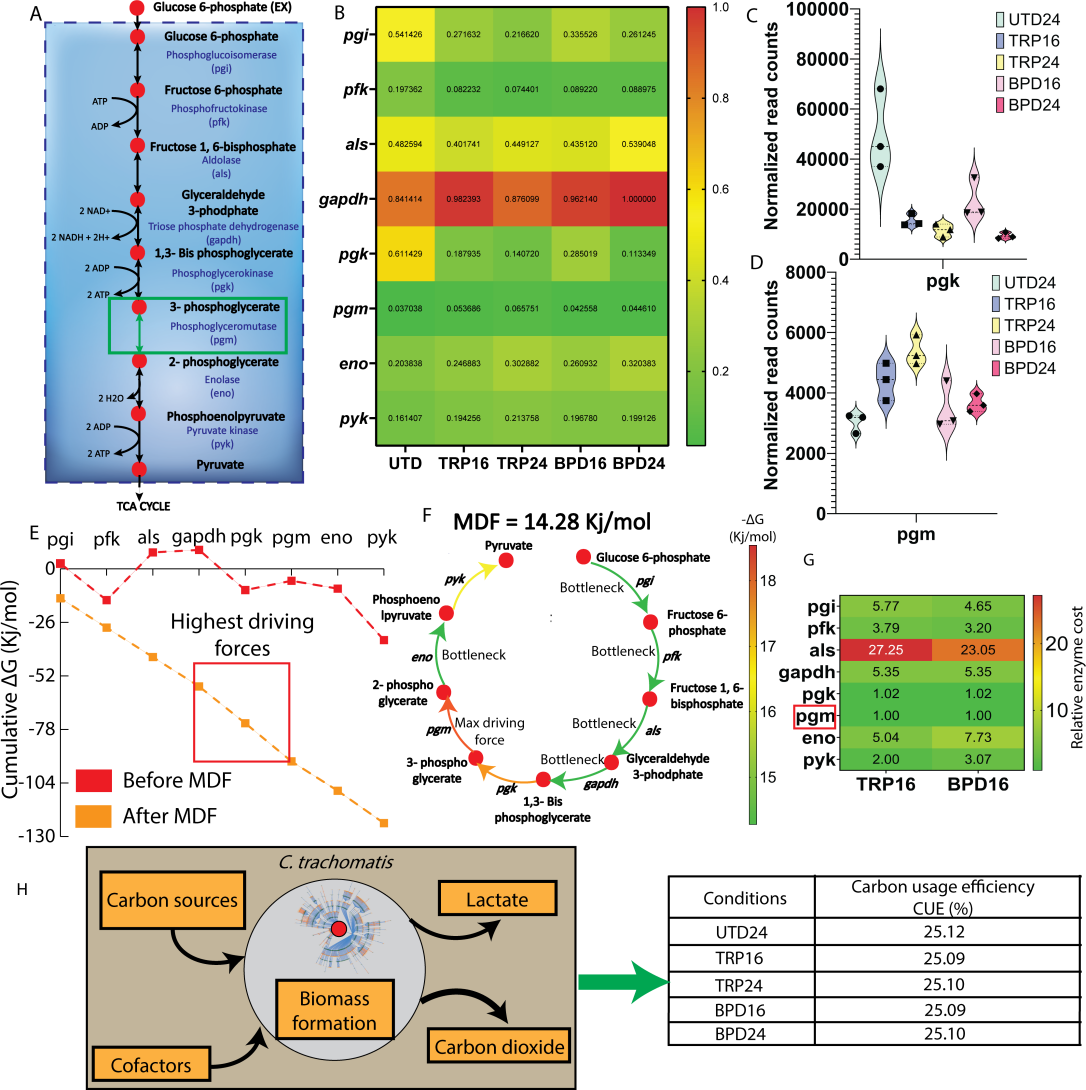
Regulatory reactions in CTL to enter persistence. (**A**) Phosphoglycerate mutase in the glycolysis pathway. (**B**) Normalized gene expression heatmap of genes involved in the glycolysis. (**C**) Transcripts level of *pgk* in TRP16-UTD24 and BPD16-UTD24. (**D**) Transcripts level of *pgm* in TRP16-UTD24 and BPD16-UTD24. (**E**) Max/min driving force (MDF) analysis showing the cumulative driving force of glycolysis. (**E**) The driving force plot showing phosphoglycerate mutase (*pgm*) and phosphoglycerate kinase (*pgk*) are two top most driving force reactions. (**F**) Individual driving force of each reactions. (**G**) The enzyme cost of glycolysis for TRP16 showing phosphoglycerate mutase has the lowest enzyme costs. (**H**) Carbon usage efficiency for UTD24, TRP16, TRP24, BPD16, and BPD24.

As CTL enters persistence, the metabolic difference between UTD24 and TRP16/BPD16 can give insight into the impact of CTL global transcriptome rewiring on its metabolism. To identify the metabolic differences between UTD24 and TRP16, we used the TRP16 model and implemented MBA (40). MBA revealed that, for TRP16, allowing phosphoglycerate mutase (*pgm; ctl0091*) from the glycolysis pathway to carry a similar reaction flux as UTD24 resulted in the same biomass growth rates for UTD24 and TRP16 conditions. The resulting reaction fluxes of other reactions of UTD24 and TRP16 were also found to be similar after making this change to *pgm* flux. This result indicates that potential regulation of *pgm* is the metabolic trait of rewiring the CTL global transcriptomics response as it enters persistence (Fig. 5A and B). The same result was obtained for the BPD16. The transcript profiles of *pgm* and the gene encoding the upstream reaction phosphoglycerate kinase (*pgk*) showed opposite expression levels under TRP16 condition, with the former being increased (Fig. 5C and D). Similar results were obtained for BPD16. However, the evidence of increased levels of transcripts does not necessarily equate to increase levels of protein in CTL during starvation conditions (6, 13).

To further dissect the significance of *pgm* transcriptional regulation in persistence, we performed max/min driving force (MDF) analysis (63) on the glycolysis pathway of CTL. MDF analysis maximizes the total driving force of a given pathway within the biologically relevant concentration of different metabolites. Figure 5E indicates the driving force of glycolysis before and after the MDF analysis. Among all the reactions in glycolysis, MDF analysis predicted that *pgm* has the highest driving force (Fig. 5F), while *pgk* has the second-highest driving force (Fig. 5F). Concentrations of different metabolites, predicted from MDF analysis, are shown in Fig. S5C. In the context of MDF, the “shadow price” (64) of a reaction accounts for the Impact of Gibb’s free energy of said reaction on the overall pathway thermodynamics. Similarly, the shadow price of concentrations for each metabolite indicates the impact of small perturbations of metabolite concentration on the overall pathway thermodynamics. Therefore, we calculated the shadow price of the driving forces of each of the reactions (Fig. S5D) and found the first three steps of glycolysis (*pgi*, *pfk*, and *als*) having the most impact on the overall driving force with changing reaction fluxes. Next, we calculated the shadow price of concentrations of each of the metabolites and found H+, glyceraldehyde 3-phosphate, and glycerone phosphate have the most impact on the overall driving force despite small changes in concentrations (Fig. S5E). Flux-force efficacy relationship indicated a high proportion of each reaction in the forward direction, and thus, the glycolysis pathway was enzymatically highly efficient (Fig. S5F). In addition, we calculated the enzyme cost (65) of each reaction from their enzyme turnover rate, $k_{cat}$ (Table S3). The turnover rates were calculated using DLKcat for CTL (66). From the enzyme cost analysis, *pgm* has the lowest enzyme costs compared to the other reactions of glycolysis, and *pgk* has the second lowest enzyme costs, indicating a low carbon investment to catalyze those reactions compared to other glycolysis reactions (Fig. 5G). To ensure the observed thermodynamics driving force and enzyme cost implications are not an artifact of reduced carbon usage efficiency (CUE) of CTL, we calculated CUE for all the conditions (67) and found that CUE remained 25% for both unstressed and stressed conditions (Fig. 5H). Therefore, it is evident that the thermodynamics and enzyme cost of *pgm*, rather than its CUE, could potentially dictate its regulatory role in CTL persistence.

Explaining unusual bacterial phenomena through thermodynamic driving force and protein cost is not uncommon in the systems biology domain. For example, the overflow metabolism is an unusual phenomenon for both *E. coli* (68) and yeast (69), which was well explained through protein cost analysis. Moreover, thermodynamic driving force analysis also explained the need for Entner–Doudoroff pathway over Embden-Meyerhof-Parnas, despite having a lower ATP yield (70). This work is also an effort to understand CTL persistence by combining thermodynamics driving force and protein cost analysis with an additional component of CRISPRi-mediated gene silencing.

### Validation of thermodynamics and enzyme cost analysis through CRISPRi-based suppression and starvation experiment

Using systems biology approaches, we predicted that *pgm* was regulated at the levels of transcription (from the MBA), thermodynamics driving force, and enzyme cost. However, other glycolysis enzymes, including *pgk*, were regulated only at the level of thermodynamic driving cost and enzyme cost. Therefore, we predicted a *pgm*-mutant would have greater impact on chlamydial growth compared to a *pgk*-mutant, as compared to the wild type.

**Fig 6:**
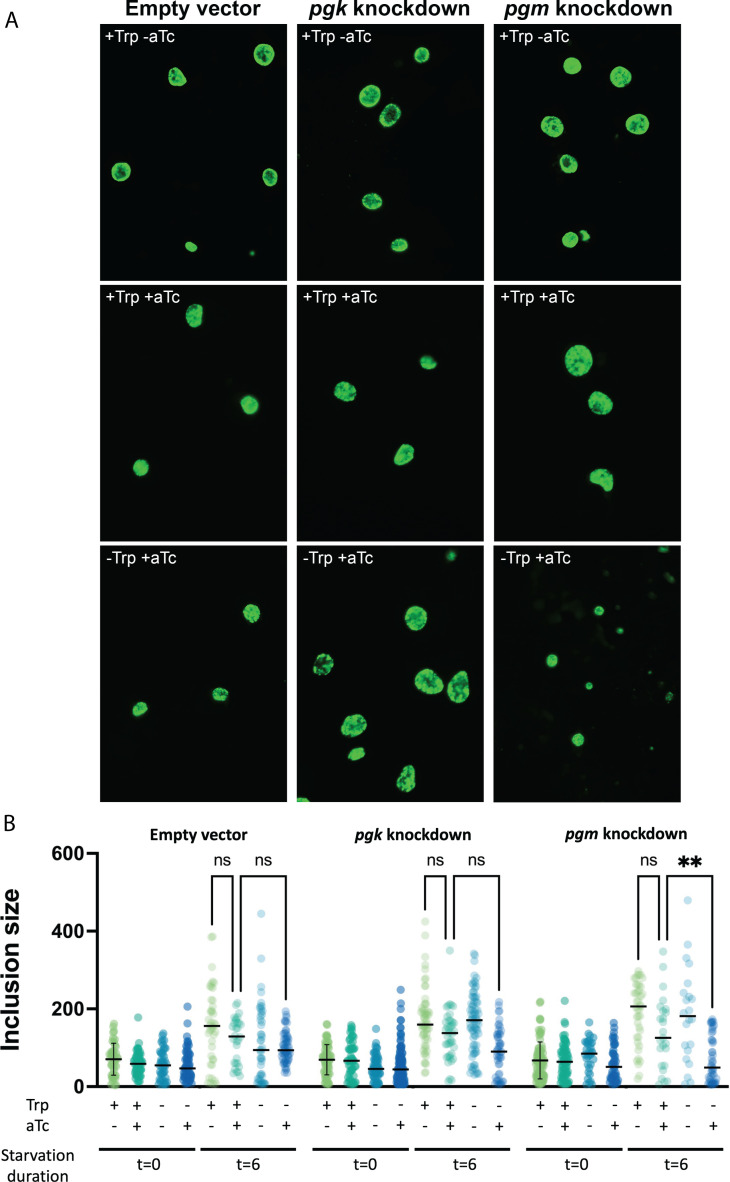
*In vitro* experiments support model prediction that *pgm* plays a major role in pushing *C. trachomatis* to persistence. (**A**) Tryptophan deprivation for 6 hours resulted in a small reduction in inclusion size. However, with pgm knockdown, the decline became more severe in tryptophan-limited and knockdown-inducing settings. (**B**) Inclusion reduction became more evident in tryptophan-limited and knockdown-inducing settings, with the pgm knockdown showing a statistically significant difference in inclusion size, corroborating the modeling studies’ findings.

To test these predictions, *C. trachomatis* inducible knockdown transformants targeting *pgk* or *pgm* were created using CRISPRi as previously described (71). In these transformants, the CRISPR RNA (crRNA) that targets the promoter region of either *pgk* or *pgm* is constitutively expressed, while the expression of an inactive Cas12 endonuclease (dCas12) is induced with anhydrotetracycline (aTc). The RNA-DNA hybrid formed by the crRNA and genomic DNA is recognized by dCas12, where it binds and blocks RNA polymerase processivity. A control *C. trachomatis* was created by transforming *C. trachomatis* with the pL12CRia plasmid that lacks a crRNA. The knockdown strains and the empty-vector control were validated for dCas12 induction by aTc, reduction in expression of the target genes, CTL development, and bacterial replication (Fig. S6A through C). In the absence of induction under normal growth conditions, all strains grew equally well (Fig. 6A).

Computational modeling indicated the importance of *pgm* in CTL entry into persistence. To test this prediction, the strains were grown in HeLa cells for 10 hours, followed by a 4-hour induction of knockdown by treatment with 5 nM aTc. The experimental groups were further subjected to either mock- or tryptophan-starvation for an additional 6 hours, with aTc maintained in the growth media. At the end of the experiment (22 hours post-infection), samples were fixed and processed for immunofluorescence staining of the chlamydial inclusions. Images were collected by confocal microscopy, and inclusion size was measured by NIH ImageJ particle analysis plug-in. Inclusion size correlates with bacterial growth and replication, in that increased inclusion volume is necessary to accommodate an increase in bacterial numbers. As shown in Fig. 6A, all strains yielded similar inclusion sizes in tryptophan-replete media without induction of knockdown (i.e., +Trp and −aTc). Tryptophan starvation for 6 hours led to a slight reduction in inclusion size, as expected. However, the inclusion size reduction became more pronounced in tryptophan-limited and knockdown-inducing conditions (i.e., −Trp and +aTc), with the *pgm* knockdown exhibiting a statistically significant difference in inclusion size (Fig. 6B), thus validating the results obtained from the modeling studies. Collectively, these data implicate *pgm* as a critical determinant of sensitivity to tryptophan starvation-mediated persistence. Moreover, this experimental validation also justifies the use of a template Gram-negative bacteria biomass equation in the absence of a CTL-specific biomass equation to model CTL metabolism.

### Cellular objective of global stress response

**Fig 7:**
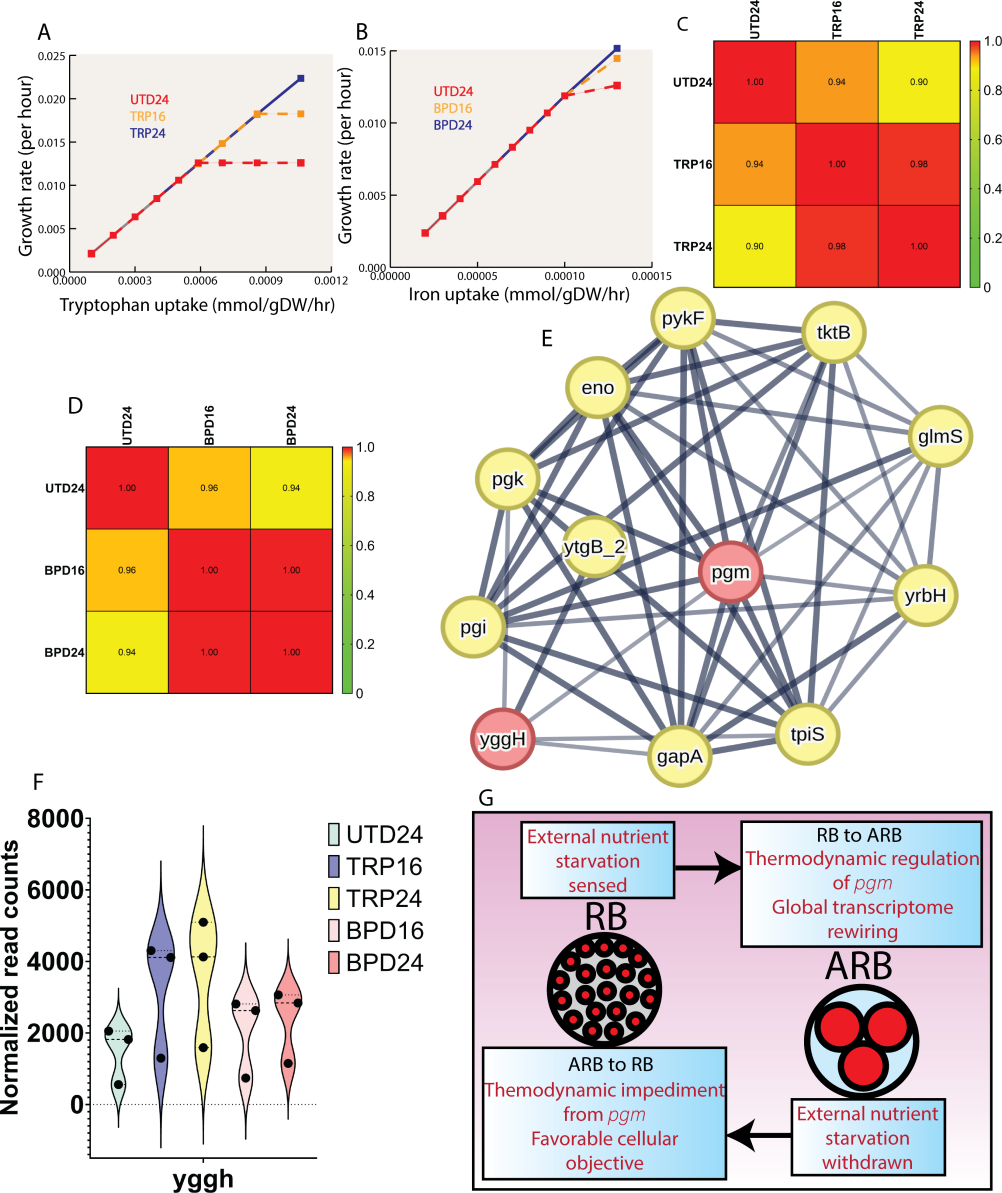
Reaction halting CTL to undergo secondary differentiation to EB. (**A**) The growth rate vs tryptophan uptake (UTD24, TRP16, and TRP24). (**B**) The growth rate vs iron uptake (UTD24, BPD16, and BPD24). (**C**) The correlation matrix calculated among growth rates of UTD24, TRP16, and TRP24 using 95% CI and a two-tailed test. (**D**) The correlation matrix calculated among growth rates of UTD24, BPD16, and BPD24 using 95% CI and a two-tailed test. (**E**) Protein interaction map for the *pgm* enzyme. The minimum required interaction score was 0.50, and top five interactions allowed for first shell. (**F**) Normalized read counts of *yggH* in different conditions. (**G**) Schematic of the mechanism proposed to enter persistence and factors that keep CTL in the persistence mode.

Our data suggest CTL regulates *pgm* activity, in conjunction with transcriptional changes, as a consequence of exposure to nutrient limiting conditions. We next sought to investigate the cellular objective of this global transcriptome rewiring. To gain insight into the significance of metabolic rewiring associated with tryptophan starvation, we plotted biomass growth rate against different tryptophan uptake rates. If we made more tryptophan available for uptake in the case of TRP24, then it could grow at a higher growth rate compared to TRP16 and UTD24 (Fig. 7A). A similar result was obtained for BPD16 and BPD24 (Fig. 7B). We also calculated the correlation matrix of growth patterns (Fig. 7C and D) under tryptophan and iron starvation conditions. Similar to the previous analysis (Fig. 4I; Fig. S5A), stress conditions were better correlated.

However, a previous study (72) suggested that, with a shorter starvation timeline, CTL should better restore its growth and start differentiating to the RB upon availability of nutrients. In other words, it should be easier to restore the growth of CTL after 16 hours of nutrient starvation compared to 24 hours of nutrient starvation. Thus, we explored the model further to investigate this phenomenon. We again applied MBA in the TRP16 metabolic model. Surprisingly, we found that if *pgm* reaction flux was relaxed to its value obtained from TRP24 model (Fig. 5A), the biomass of TRP16 and TRP24 became the same. A similar result was obtained for the BPD16 and BPD24. This analysis indicates that the cellular objective of the CTL global transcriptome rewiring during persistence is to reach such a cellular phenotype so that, when the stress is withdrawn, CTL can exit persistence immediately and re-enter the normal developmental cycle. CTL can do that by relaxing its regulation of *pgm*, which is critical. This final objective also supports the evolutionary selection of CTL, which is to maximize its own fitness function. The protein interaction network suggests that *pgm* has a strong interaction with *yggH* (Fig. 7E). YggH catalyzes the *S*-adenosyl-l-methionine-dependent formation of *N*^7^-methylguanosine at position 46 (m^7^G46) in tRNA and can impact the activity of tRNA (73). It was previously reported that inhibition of tRNA synthetase activity can induce persistence in CTL (74). The normalized read counts of *yggH* show significantly higher levels of transcripts in all the stress conditions compared to the UTD24 (Fig. 7F). Thus, the regulation of *pgm* may be mediated in part by changes in *yggH*. The proposed mechanism that led CTL to enter persistence is shown in Fig. 7G. Quantitative proteomics from the literature (75) indicated that the ARB is primed for a burst in metabolic activity upon nutrient availability, whereas the RB is geared toward nutrient utilization, a rapid increase in cellular mass, and securing the resources for an impending transition into the EB form. In addition to confirming previously published findings, this study indicates that *pgm* may be a mediator of ARB-to-RB transition.

This study utilized transcriptomics data sets and systems biology tools, including K-means clustering and GSM, to analyze the transcriptome and metabolism of CTL under tryptophan and iron stress conditions. The findings suggest a global transcriptomic rewiring in CTL under stress, impacting its persistence mechanisms. Through a combined systems and synthetic biology approach, the study identified *pgm* as a regulator of CTL persistence, highlighting the importance of systems biology in understanding the growth and development of the obligate intracellular pathogen CTL, complementing insights gained through molecular genetics approaches in model prokaryotes. Future research efforts will be geared toward studying the interaction between CTL and different host epithelial cells (e.g., endocervical and endovaginal epithelial cell). Similar to this study, we aim to identify the metabolic signatures that influence the CTL infection in these cells.

## MATERIALS AND METHODS

### Cell lines

Human female cervical epithelial adenocarcinoma HeLa cells (RRID: CVCL_1276) were cultured at 37°C with 5% atmospheric CO_2_ in Dulbecco’s Modified Eagle Medium (DMEM; Gibco, Thermo Fisher Scientific) supplemented with 10  µg/mL gentamicin, 2  mM l-glutamine, and 10% (vol/vol) filter sterilized fetal bovine serum. For all experiments, HeLa cells were cultured between passage numbers 3 and 15. HeLa cells were originally authenticated by ATCC via STR profiling and isoenzyme analysis per ATCC specifications.

### Bacterial strains

*C. trachomatis* serovar L2 434/Bu was originally obtained from Dr. Ted Hackstadt (Rocky Mountain National Laboratory, NIAID). Chlamydial EBs were isolated from infected HeLa cells at 36–40 hpi and purified by density gradient centrifugation essentially as described (76). For infections, at 80%–90% confluence, HeLa cells were first washed with Hanks Buffered Saline Solution (HBSS; Gibco, Thermo Fisher Scientific), and ice-cold inoculum prepared in HBSS at the indicated multiplicity of infection (MOI) was overlaid onto the cell monolayer. To synchronize the infection, inoculated cells were then centrifuged for 15 minutes at 500× relative centrifugal force (RCF), 4°C in an Eppendorf 5810 R tabletop centrifuge with an A-4–81 rotor. The inoculum was then aspirated, and pre-warmed DMEM (or relevant media with treatment supplementation) was added to the cells. Infected cultures were then returned to the tissue culture incubator until the indicated time post-infection.

### Genome copy number quantification

All quantitative PCR (qPCR) assays were performed using Power Up SYBR Green Master Mix (Applied Biosystems, Thermo Fisher Scientific) essentially as previously described (77, 78). In brief, cDNA was diluted 1:5–1:10, and gDNA was diluted 1:50–1:100 in nuclease-free H_2_O (dilutions were identical within each experiment). The 2× PCR master mix was diluted to 1× in nuclease-free H_2_O with specific primers to the euo open-reading frame diluted to 500 nM. To 79 µL of the master mix solution, 3.3 µL of template (cDNA or gDNA) was added and then aliquoted into three 25 µL technical replicate reactions in a 96-well optical plate. Reactions were analyzed on a QuantStudio 3 real-time PCR system with standard SYBR cycling conditions. All assays were performed with a melt-curve analysis to ensure specific product amplification across samples. Primer sets used in qPCR were validated against a standard curve of C. trachomatis L2 gDNA diluted from 2 × 10–3 to 2 × 100 ng per reaction. Ct values generated from each experimental reaction were then fit to a standard curve, and only primer sets with an efficiency of 100% ± 5% were used. The sequences of the forward and reverse primers are as follows:

euo forward – 5’- GCTGTTCCTGTTACTTCGCAAA - 3’

euo reverse – 5’- AACATAGATAGCCTGACGAGTCACA – 3’

GEs were calculated by first converting the mean Ct of the triplicate technical replicate reactions to a ng quantity of gDNA (ng template) with the linear equation generated from the standard curve of the euo primer pair. This value was then normalized to the total ng/μL gDNA isolated for each sample.

### Validation of CRISPRi knockdown strains

The pBOMBL12Cria::L2 empty vector was digested with BamHI and treated with alkaline phosphatase (ThermoFisher). Two nanogram of the crRNA gBlock (IDT DNA, Coralville, IA) targeting the intergenic region of either *pgm* or *pgk* (Table S4) was used in a HiFi reaction with 25 ng of digested plasmid according to the manufacturer’s instructions [New England Biolabs (NEB); Ipswich, MA]. Twenty-five microliter of chemically competent *E. coli* 10β cells (NEB) were transformed with 2 µL of the HiFi reaction and plated on Luria-Bertani (LB) agar with ampicillin selection. Plasmid was isolated from overnight cultures of individual colonies grown in LB broth with ampicillin and verified by restriction digest and sequencing. Two microgram of verified plasmid was used to transform *C. trachomatis* serovar L2 (-pL2) lacking its endogenous plasmid as described elsewhere (71). After obtaining transformants carrying either the pBOMBL12Cria(*pgm*)::L2 or pBOMBL12Cria(*pgk*)::L2 CRISPRi vectors, cells were infected with an MOI of 1. At 10 hpi, the expression of the dCas12 protein was induced or not with 2 nM aTc, and RNA and DNA samples were collected and processed as described previously at 10, 14, and 24 hpi (71). Transcript levels for *pgm* and *pgk* were determined by RT-qPCR from equal volumes of cDNA using gene-specific primers (Table S4) on a QuantStudio3 (Applied Biosystems; Thermo) and normalized to genomic DNA levels determined from equal masses of DNA by qPCR using the *pgm* qPCR primer set. A standard amplification cycle with a melting curve analysis was used for the qPCR analysis and compared to a standard curve of *C. trachomatis* L2 genomic DNA. Data are representative of two biological replicates assessed in triplicate.

### Treatment conditions and induction of knockdown of *pgk* and *pgm* transcription by CRISPRi

HeLa cells were infected at an MOI of 0.5 (*t* = 0 hpi) and were maintained in complete DMEM for 14 hours. The infected cells were then exposed to anhydrous tetracycline (aTc) at 5 nM for 4 hours to induce knockdown of expression (*t* = 14 hpi). Tryptophan depletion was performed at *t* = 18 hpi by first washing cells with HBSS and then replacing complete DMEM with tryptophan-depleted DMEM-F12 (U.S. Biological Life Sciences). Treated cells were then returned to the tissue culture incubator for the remainder of the experimental time course. At *t* = 22 hpi, samples were collected and fixed with freshly prepared 4% paraformaldehyde and permeabilized for 10 minutes with 0.1% Triton X-100 in phosphate-buffered saline (PBS). Samples were immunostained with mouse antibody against the *C. trachomatis* major outer membrane protein (1:1,000 dilution). Samples were incubated at 4°C overnight with constant rocking. The next day, the primary antibody solution was removed, and samples were rinsed 3× with 1× PBS and incubated with 1:1,000 dilution of goat anti-mouse IgG conjugated with Alexa-488 for 1 hour. Samples were rinsed and visualized by fluorescence microscopy. Images were processed, and inclusion size was measured using NIH ImageJ.

### Genome-scale metabolic model reconstruction of CTL

The genome-scale metabolic network reconstruction was based on available genome information of the model strain *C. trachomatis* L2 434/Bu (KBase Genome ID: GCF_000068585.1), according to the ModelSEED databases (79), BRENDA (80), and available literature. Additional reactions were added to the model based on the previously published genome-scale metabolic model of CTL (30), experimental data (8), and previously published literature on metabolic traits of CTL. Gap filling for pathways in our model was first conducted in Kbase (41) and then by OptFill (44) based on the complete media, as complete media was used in the experimental settings as well to grow CTL *in vitro*. In total, 95 reactions were added to the draft model to fill the gaps. The model was further checked for elemental mass balance, and MEMOTE reports confirm that all the reactions in the model are mass balanced. gene-protein-reaction association (GPR) for all the reactions was manually curated from the KEGG database. As a result, 525 out of 692 reactions have a GPR relationship. We used the General Algebraic Modeling System (GAMS) platform along with the CPLEX solver for solving all the optimization problems. NEOS server can be used to run GAMS codes without having to buy the license. Details of the procedure of running GAMS codes in NEOS server can be found in the literature (81). However, for the convenience of COBRApy users, SBML version of the iCTL278 is also provided.

### Parsimonious flux balance analysis

pFBA (21) is constrained based optimization technique to model GSMs. The pseudo-steady state mass balance in pFBA is represented by a stoichiometric matrix, where the columns represent metabolites, and the rows represent reactions. For each reaction, upper and lower bounds are imposed based on thermodynamic information. pFBA provides the flux value for each reaction in the model according by solving the following optimization problem:


$$\begin{array}{c} \max v_{\text{biomass }}-0.0001\sum_{j\in J}\left|v_{j}\right|\\ Subject\;to:\\ \sum_{j\in J}S_{ij}v_{j}=0,\forall i\in I\;\;\;[1]\\ a_{j}\leq v_{j}\leq b_{j}\;\;[2] \end{array}$$

In this formulation, I is the set of metabolites, and J is the set of reactions in the model. $S_{ij}$ is the stoichiometric matrix with i indicating metabolites and j indicating reactions, and $v_{j}$ is the flux value of each reaction. The objective function, $v_{biomass}$, is the proxy of the growth rate of an individual cell. $a_{j}$ and $b_{j}$ are the lower and upper bounds of flux values for each reaction. For forward reactions, the highest possible bounds were 0 mmol/gDW/h to 1,000 mmol/gDW/h. For the reversible reactions, the highest possible bounds were −1,000 mmol/gDW/h to 1,000 mmol/gDW/h.

### Flux variability analysis

The result from FBA may include degenerate optimal solutions, this FVA (20) was used to find out the alternate flux distributions. The formulation is the following:


$$\begin{array}{c} max/min\;\;v_{j}\;\;\;\;\;\;\;\;\;\;\;\;\;\;\;\;\;\;\;\;\;\;\;\;\;\;\;\;\;\;\;\;\;\;\;\;\;\;\;\;\;\;\;\\ Subject\;\;to:\\ \sum_{j\in J}S_{ij}v_{j}=0,\;\forall i\in \;I\;\;\;\;\;\;\;\;\;\;\left[3\right]\\ v_{biomass}=v_{biomass}^{max}\;\;\;\;\;\;\;\;\;\;\;\;\;\left[4\right]\\ a_{j}\leq v_{j}\leq b_{j},\;\;\;\forall j\in J\;\;\;\;\;\;\;\;\;\;\;\left[5\right] \end{array}$$

FVA maximizes and minimizes each of the reaction fluxes subject to the pseudo-steady mass balance, fixing biomass growth rate for a specific condition, and upper and lower bounds on reaction fluxes. In this manuscript, all the FVA resulted in very tight bounds (changes can only be observed after two decimal place).

### E-Flux algorithm

E-Flux is an extension of FBA/pFBA that uses transcriptomic data to further constrain the feasible space based on the transcriptomics data (38). The E-flux algorithm involved solving the following linear optimization problem:


$$\begin{array}{c} \max\;\;\;v_{biomass}-0.0001\sum_{j\in J}|v_{j}|\\ Subject\;to:\\ \sum_{j\in J}S_{ij}v_{j}=0,\;\forall i\in \;I\;\;\;\;\;\;\;\;\;\;\;\;\;\;\;\;\;\;\;\;\left[6\right]\\ a_{j}\leq v_{j}\leq b_{j}\;\;\;\;\;\;\;\;\;\;\;\;\;\;\;\;\;\;\;\;\;\;\;\;\;\;\;\;\;\;\;\;\;\;\;\;\left[7\right] \end{array}$$

where $a_{j}$ and $b_{j}$ are the minimum and maximum allowed fluxes through reaction j, based on the transcriptomics data. The E-Flux method calculates the upped bound, $b_{j}$, for the $j^{th}$ reaction according to the following function of the gene expression:


$$b_{j}=\left(expression\;level\;of\;genes\;associated\;with\;reaction\;j\right)\;\left[8\right]$$

In this manuscript, $b_{j}$ is the exact level of each reaction was calculated through its GPR association [i.e., based on “OR” (addition of gene expressions) and “AND” (minimum of gene expressions) relation]. If the reaction catalyzed by the corresponding enzyme was reversible then $a_{j}=-b_{j}$, otherwise $a_{j}=0$.

### Flux sum analysis

The metabolite pool size of ATP in the validation section was determined based on the FSA method (33). The flux sum is a measure of the amount of flux through the reactions associated with either the production or consumption of the metabolite. The range of the flux sum can be calculated as follows:


$$\left[\begin{array}{c} Max/Min\;\;0.5\sum_{j=1}^{m}\left|S_{ij}v_{j}\right|\;\\ Subject\;\;to:\\ \sum_{j=1}^{m}S_{ij}v_{j}=0,\;\;i\in I\;\;\;\;\;\;\;\;\;\;\;\;\;\left[9\right]\\ a_{j}\leq v_{j}\leq b_{j}\;\;\;\;\;\;\;\;\;\;\;\;\;\;\;\;\;\;\;\;\;\;\;\;\;\;\;\;\left[10\right]\\ v_{biomass}=v_{biomass}^{max}\;\;\;\;\;\;\;\;\;\;\;\;\;\left[11\right] \end{array}\right],\;\forall i\in I$$

Here, set I represents the set of metabolites for which the flux sum will be calculated. By linearizing the objective function, the resulting formulation became a mixed-integer linear programming problem. Therefore, the basic idea was to determine the range of the flux-sum of a metabolite under a given condition by fixing the maximum biomass growth of that condition ( ).

### K-mean clustering

K-mean clustering algorithm was used to classify different genes into different clusters. The number of clusters was determined using the Elbow method (Fig. S7). The whole K-mean clustering was implemented in Python, using numpy, pandas, and sklearn modules. Different plots for the K-mean clustering were generated using matplotlib module. Default setting of K-mean clustering, mentioned in the sklearn, was not changed in this study.

### Metabolic bottleneck analysis

To determine the metabolic bottleneck in a GSM, metabolic bottleneck analysis (40) was used. The formulation is as follows:


$$\left[\begin{array}{c} Max\;\;v_{biomass}\\ Subject\;\;to:\\ \sum_{j=1}^{m}S_{ij}v_{j}=0,\;\;i\in I\;\;\;\;\;\;\;\;\;\;\;\;\;\;\;\;\;\;\;\;\;\;\;\;\;\;\;\left[12\right]\\ a_{j}\leq v_{j}\leq b_{j},\;j/\left\{j^{\prime }\right\}\;\;\in \;J\;\;\;\;\;\;\;\;\;\;\;\;\;\;\;\;\left[13\right]\\ v_{j^{\prime },min,}\leq v_{j^{\prime }}\leq v_{j^{\prime },max},\;j^{\prime }\;\;\in \;J\;\;\;\;\;\left[14\right] \end{array}\right],\;\forall j^{\prime }\in J$$

Here, $a_{j}$ is the lower bound reaction $v_{j}$, and $b_{j}$ is the upper bound of reaction $v_{j}$. Both $a_{j}$ and $b_{j}$ were calculated from the transcriptomics data and gene-protein-reaction association. $v_{j^{`},min}$ is the expanded lower bound of the reaction $j^{`}$, and $v_{j^{`},max}$ is the expanded upper bound of the reaction $j^{`}$. In this case, we set $v_{j^{`},min}=-1000\frac{mmol}{gDW.hr}$ and $v_{j^{`},max}=1000\frac{mmol}{gDW.hr}$. We solved the optimization problem by maximizing the biomass $v_{biomass}$ for the new expanded flux space of each reaction $j^{`}$ in an iterative manner and then recorded the biomass growth rate. From this biomass growth rate collections, we can check for which $j^{`}$ biomass growth rate increased significantly. Then that $j^{`}$ can be considered as the metabolic bottleneck of a given metabolic network.

### Max/min driving force analysis

To find out the thermodynamic driving force and thermodynamic bottleneck of a given pathway, we used MDF analysis from the literature (63). In this method, we maximized the driving force of each reaction in a given pathway within the biologically relevant concentration and found the maximum possible driving force of a pathway. The formulation of the MDF analysis is given below:


$$\begin{array}{c} axB\\ Subjectto:\\ -G\geq B\;[15]\\ G=(G^{0}+RT\cdot S^{T}\cdot x)\;[16]\\ ln(C_{m}in)\leq x\leq ln(C_{m}ax)\;[17]\\ x_{A}TP\geq 10x_{A}DP\;[18]\\ x_{N}ADH\geq 0.1x_{N}AD\;[19]\\ x_{A}DP=0.4\;[20]\\ x_{N}AD=6.5\;[21]\\ x_{3}PGA=13.0\;[22] \end{array}$$

$G^{0}$ is the standard Gibbs free energy, R is the gas constant, T is the temperature, and xandC indicates concentration. This analysis is particularly useful when metabolomics information is not fully available for a given pathway. Thus, a biologically relevant metabolite concentration range can be used to infer information about a pathway, whether it will be thermodynamically feasible or not. Furthermore, this analysis will indicate reaction(s) with the highest and lowest thermodynamic driving forces and will also provide a concentration of metabolites to support the maximum pathway driving force. Here, we fixed the ATP/ADP and NADF/NAD ratio as mentioned in the original MDF article (63). Besides concentrations (mM) of ADP, NAD, and 3-PGA were fixed in the MDF optimization problem based on literature evidence (82, 83). Shadow price of different constraints was calculated using the built-in features of GAMS. The temperature in the optimization problem was set to 37°C, which is similar to the temperature of the growth culture. The ratio of forward-to-backward reaction fluxes for each of the glycolysis reactions was calculated using the following equation.


$$\frac{v^{+}-v^{-}}{v^{+}+v^{-}}=\;\frac{e^{-\frac{\Delta G}{RT}}-1\;}{e^{-\frac{\Delta G}{RT}}+1\;}\;\left[23\right]$$

Here, ∆G was calculated from the MDF analysis. Also, $v^{+}$ and $v^{-}$ indicate reaction flux in forward and backward directions, respectively.

### Enzyme cost calculation

To find out enzyme synthesis costs for different enzymes of a given pathway, we used the following equation from the literature (65) assuming all the enzymes are fully saturated.


$$v\;=\;E\cdot \;k_{cat}\left(1-e^{-\frac{\Delta G}{RT}}\right)\;\left[24\right]$$

Here, v is the reaction flux obtained from the contextualized GSM, E is the enzyme cost, $k_{cat}$ is the enzyme turnover rate, ΔG is the Gibbs free energy obtained from the MDF analysis, R is the gas constant, and T is the temperature. Relative enzyme cost was calculated using the following equation.


$$E_{i,\;relative}\;=\;\frac{E_{i}}{E_{lowest}}\;\left[25\right]$$

Here, $E_{i,relative}$ is the relative cost of an enzyme in a given pathway, $E_{i}$ is the actual cost of an enzyme in a given pathway, and $E_{lowest}$ is the lowest cost of an enzyme in a given pathway.

### Simulation platform

The GAMS version 24.7.4 with IBM CPLEX solver was used to run pFBA and FVA, E-Flux, FSA, MBA, and MDF algorithms on the model. Each of the algorithms was scripted in GAMS and then run on a Linux-based high-performance cluster computing system at the University of Nebraska-Lincoln. Furthermore, flux sampling, tSNE plot, and K-mean clustering analysis of transcriptomics data were performed in Python using an Intel(R) Core(TM) i5-8250U CPU 1.60 GHz HP laptop with 8.00 GB of RAM and 64-bit operation with Windows 11 Home operating system.

## Data Availability

The data that support for the findings of this study can be found in the related cited articles and/or in the supplementary data. All the codes used to generate these results can be accessed in the GitHub repository (https://github.com/ssbio/c_trachomatis).

## SUPPLEMENTAL MATERIAL

Text S1 msystems.00717-24-s0001.html MEMOTE report of iCTL278.

Table S1 msystems.00717-24-s0002.xlsx Biomass sensitivity analysis.

Table S2 msystems.00717-24-s0003.xlsx Details about each cluster.

Table S3 msystems.00717-24-s0004.xlsx Details of k_cat for each reaction in glycolysis.

Table S4 and Supplemental Figures msystems.00717-24-s0005.docx List of plasmids, strains, and primers and Fig. S1–S7.

ASM does not own the copyrights to Supplemental Material that may be linked to, or accessed through, an article. The authors have granted ASM a non-exclusive, world-wide license to publish the Supplemental Material files. Please contact the corresponding author directly for reuse.
