# Supplementary material for: Metabolic model guided CRISPRi identifies a central role for phosphoglycerate mutase in Chlamydia trachomatis persistence
Source: mSystems. 2024 Jun 28;9(7):e00717-24. doi: 10.1128/msystems.00717-24 (PMC11323709; doi:10.1128/msystems.00717-24)
Supplement: Table S4 and Supplemental Figures — List of plasmids, strains, and primers and Fig. S1–S7. [file msystems.00717-24-s0005.docx]

**Metabolic Model Guided CRISPRi Identifies a Central Role for Phosphoglycerate Mutase in *Chlamydia trachomatis* Persistence**

Niaz Bahar Chowdhury ^1^, Nick Pokorzynski ^2, #^, Elizabeth A. Rucks ^2^, Scot P. Ouellette ^2^, Rey A. Carabeo ^2^, & Rajib Saha ^1, *^

^1^ Chemical and Biomolecular Engineering, University of Nebraska-Lincoln, Lincoln, Nebraska, 68508, USA.

^2^ Department of Pathology, Microbiology, and Immunology, University of Nebraska Medical Center, Omaha, Nebraska, 68198, USA.

^#^ Present address: Department of Microbial Pathogenesis, Yale University School of Medicine, New Haven, Connecticut, 06510, USA.

Running Title: *Pgm* as a Regulator of Chlamydial Persistence

^*^Address correspondence to Rajib Saha, rsaha2@unl.edu

**Supplemental information**


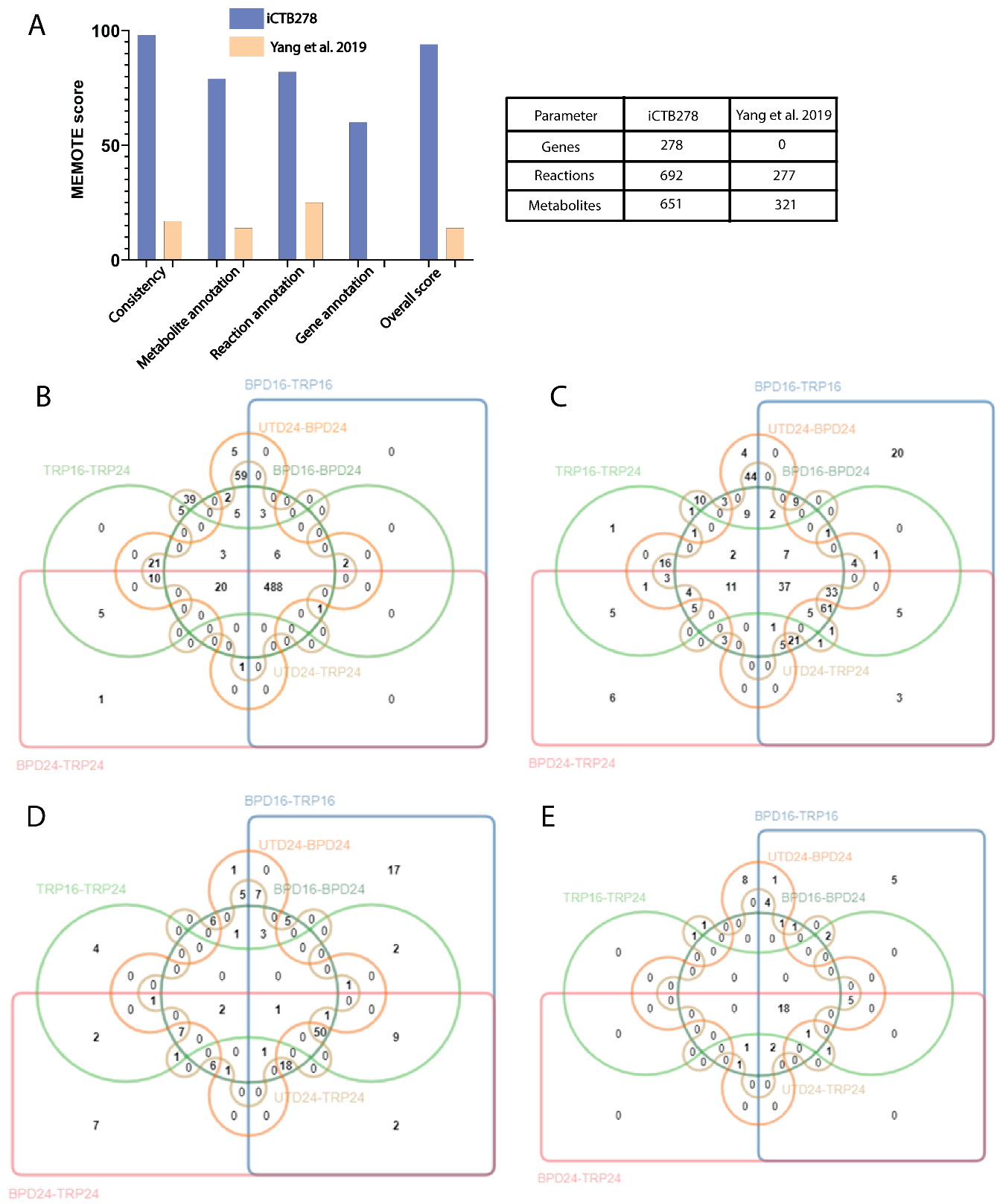


**Fig S1**. A) Comparison between *i*CTB278 and Yang et al. 2019;.B) Venn diagram to find 488 common genes present across the green clusters; C) Venn diagram to find 37 common genes present across the yellow clusters; D) Venn diagram to find 1 common genes present across the blue clusters; E) Venn diagram to find 18 common genes present across the red clusters.


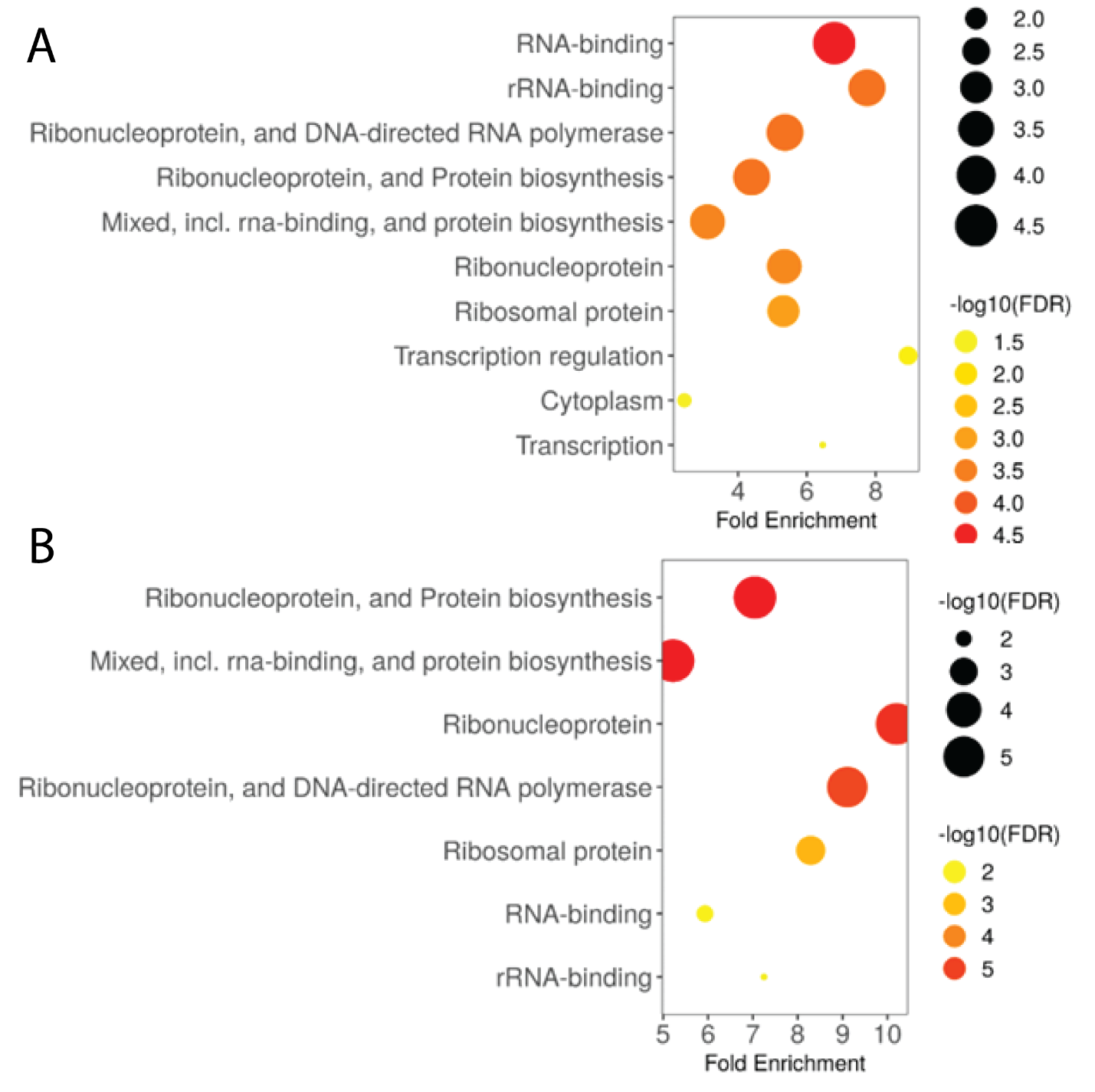


**Fig S2**. A) GO enrichment analysis for all the common genes present across the yellow clusters; B) GO enrichment analysis for all the common genes present across the red clusters.


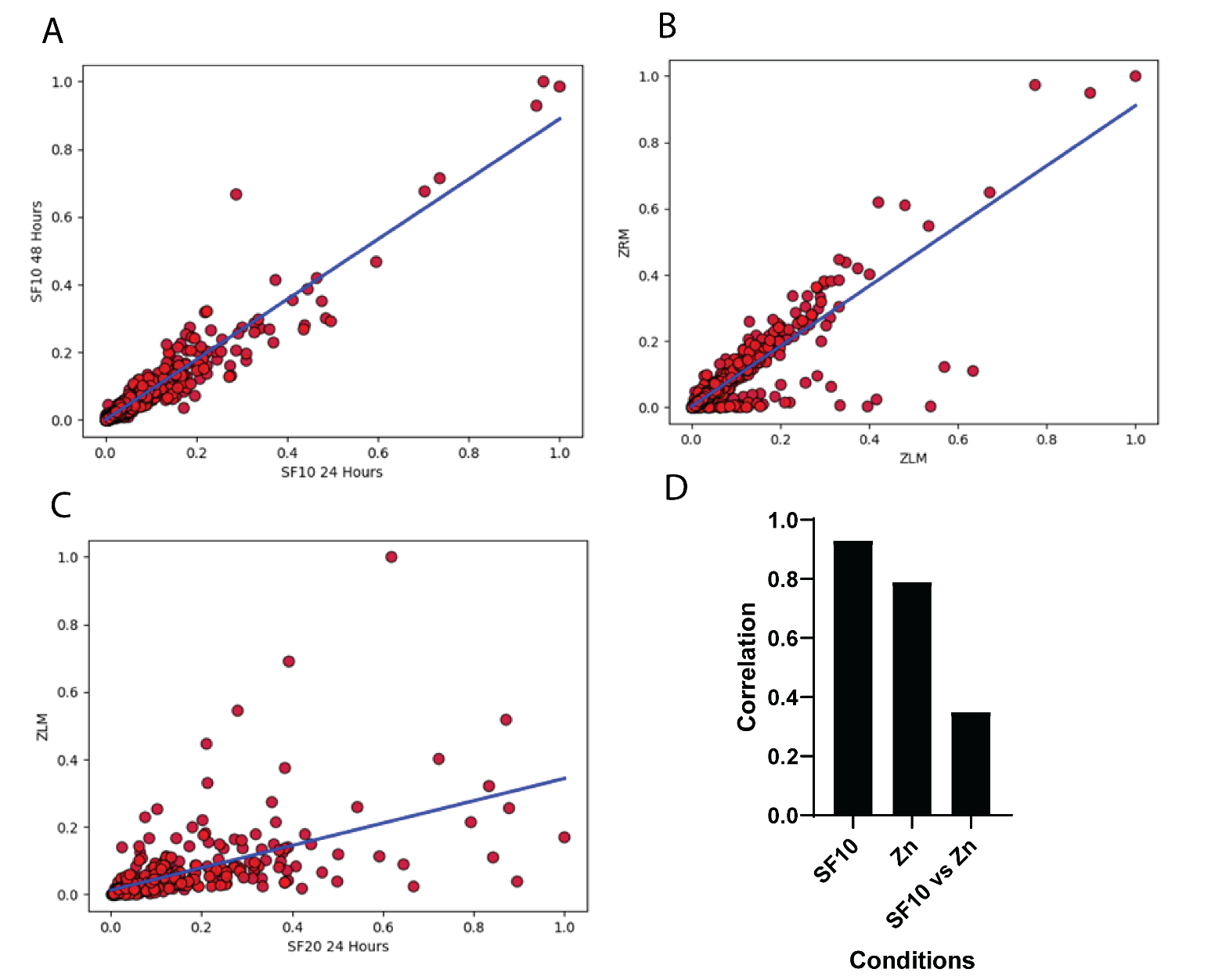


**Fig S3**. A) Scatter plot of *M. tuberculosis* exposed to SF10 lysosomal fluids for 48 hours and 24 hours; B) Scatter plot of *M. tuberculosis* exposed to Zn repleted and Zn starved medium; C) Scatter plot of *M. tuberculosis* exposed to Zn starved medium and 24 hours of SF20 lysosomal fluid; D) Correlation for different *M. tuberculosis* stress response.


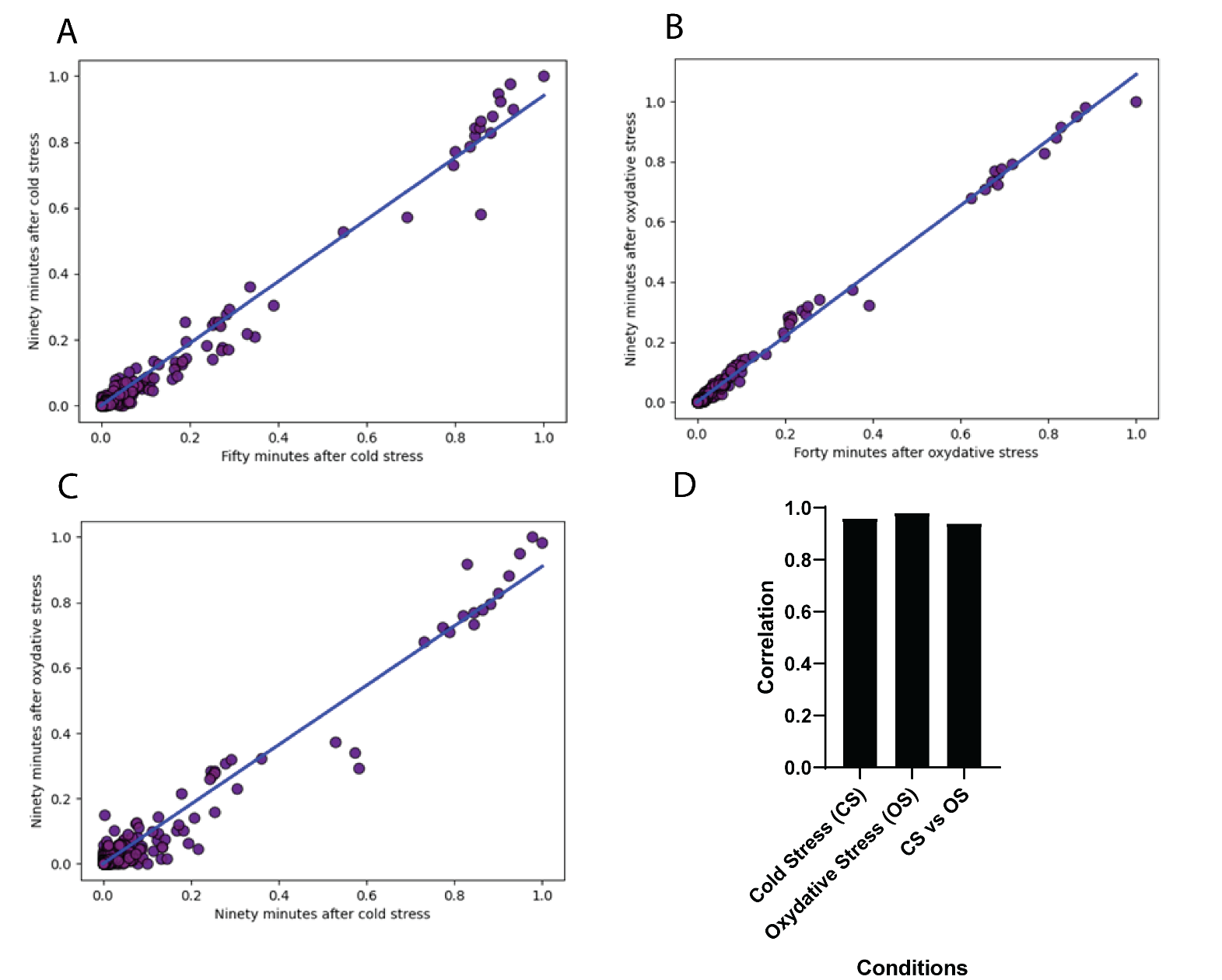


**Fig S4**. A) Scatter plot of *E. coli* exposed to ninety minutes of cold stress and fifty minutes of cold stress; B) Scatter plot of *E. coli* exposed to ninety minutes after oxidative stress and forty minutes of oxidative stress; C) Scatter plot of *E. coli* exposed to ninety minutes after oxidative stress and ninety minutes of cold stress; D) Correlation for different *E. coli* stress response.


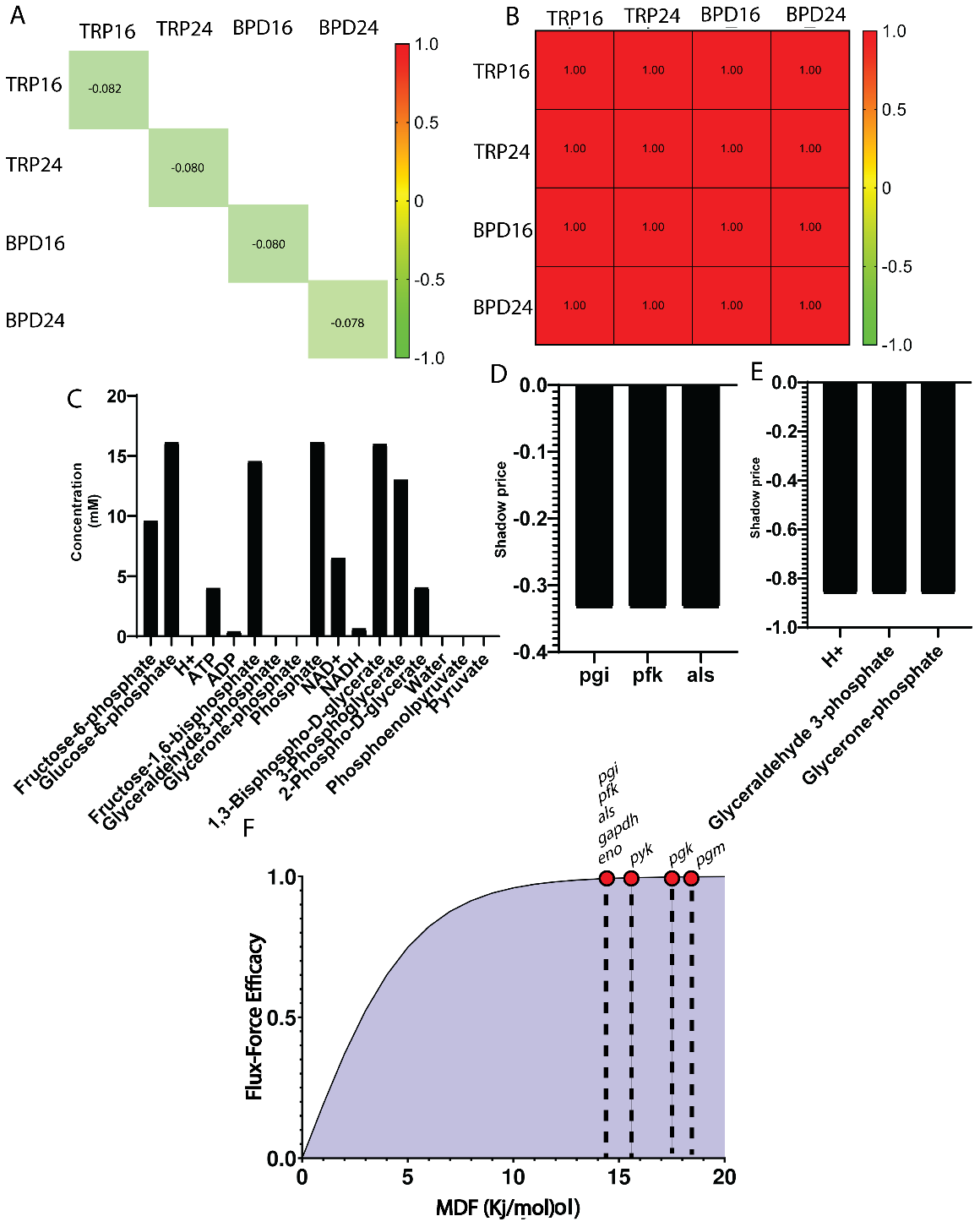


**Fig S5.** A) Correlation between transcriptomics data predicted fluxes and fluxes from *i*CTB278; B) Correlation among different conditions based on reaction fluxes from *i*CTB278; C) Concentration of different metabolites obtained from the MDF Analysis; D) Shadow price of different reactions on the overall driving force of glycolysis; E) Shadow price of concentration of different metabolites on the overall driving force of glycolysis; F) Flux-force efficacy of glycolysis pathway.


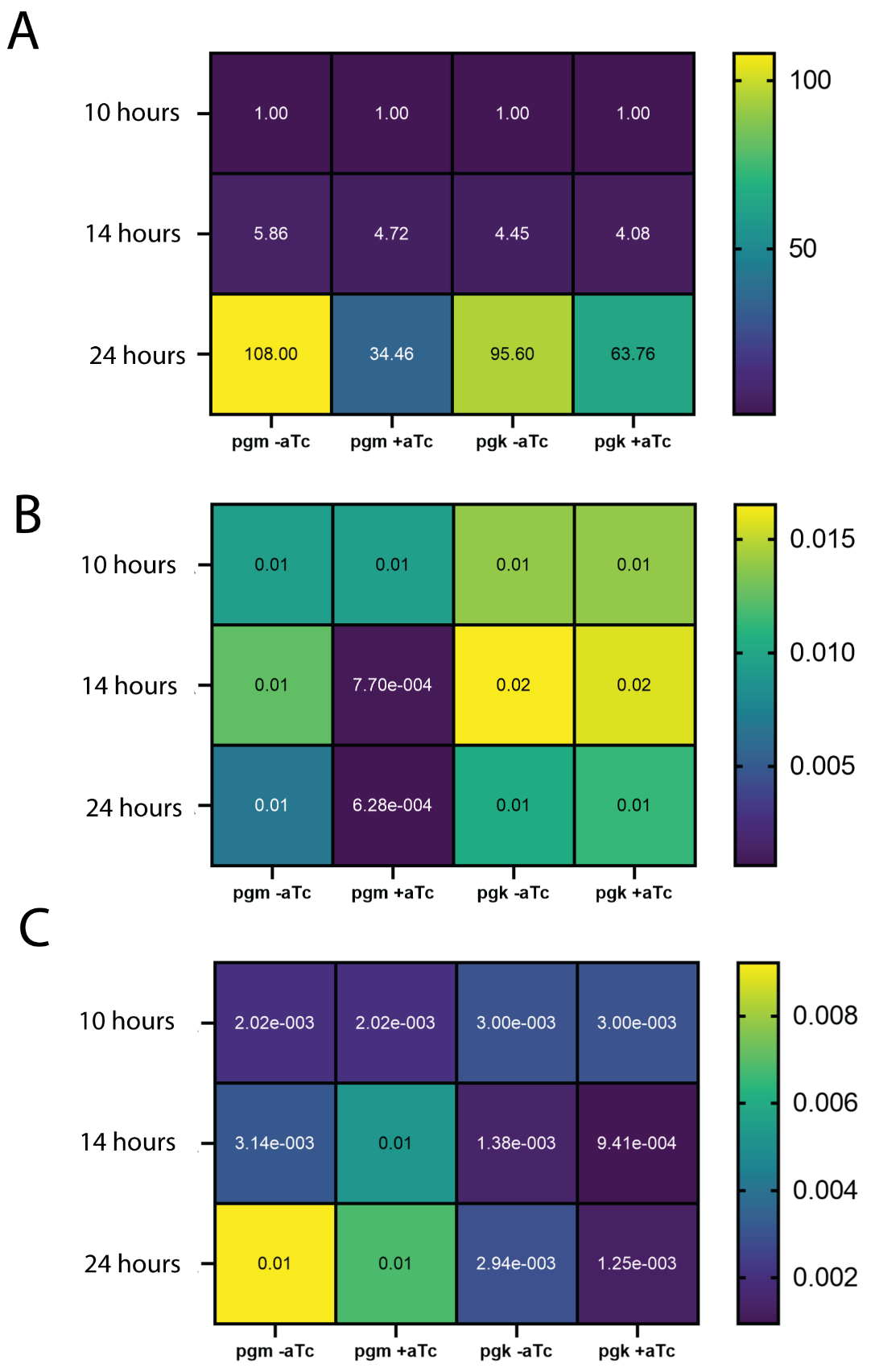


**Fig S6**. A) Relative gDNA for both the mutants with and without aTc induction; B) Gene expression profile of *pgm* for both the mutants with and without aTc induction; C) Gene expression profile of *pgk* for both the mutants with and without aTc induction.


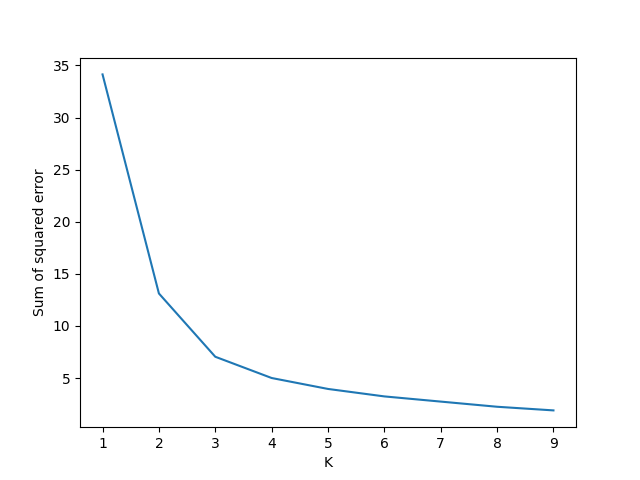


**Fig S7**. Sum of squared error was plotted against different numbers of cluster. From the graph it is evident that, when the number of clusters were four, the sum of squared error became a horizontal line. Thus we choose the number of clusters to before. Same number of clusters were obtained for all the simulations.

**Table S4 Table: List of Plasmids, Strains, and Primers**

| ***E. coli* strain** | **Relevant genotype** | **Source of reference** |
| --- | --- | --- |
| DH10β | *Δ(ara-leu) 7697 araD139 fhuA ΔlacX74 galK16 galE15 ϕ80dlacZΔM15 (e14-) recA1 relA1 endA1 nupG rpsL (*Str^R^*) rph spoT1 Δ(mrr-hsdRMS-mcrBC)* | New England BioLabs |

| **Plasmid** | **Relevant genotype** | **Origin** | **Source of reference** |
| --- | --- | --- | --- |
| pBOMBL12CRia(e.v.)::L2 | *bla* P_Nm_::*gfp* P_tet_::As_dCas12vaa | pUC19 | Ouellette et al., 2021 |
| pBOMBL12CRia(*pgm*)::L2 | *bla* P_Nm_::*gfp* P_tet_::As_dCas12vaa P*_dnaKmut_*::As_crRNA_*pgm* | pUC19 | This study |
| pBOMBL12CRia(*pgk)*::L2 | *bla* P_Nm_::*gfp* P_tet_::As_dCas12vaa P*_dnaKmut_*::As_crRNA_*pgk* | pUC19 | This study |

| **Primer name** | **Sequence** | **Features** | **Usage** |
| --- | --- | --- | --- |
| ct722 *pgm* qPCR F | CAATGACTAACCACAGCTCTCA | Forward qPCR primer | For qPCR of *pgm* |
| ct722 *pgm* qPCR R | AGGGATCATCTGCTCCATTTC | Reverse qPCR primer | For qPCR of *pgm* |
| ct693 *pgk* qPCR F | GCTATTGAGCTTGCTGCTTATG | Forward qPCR primer | For qPCR of *pgk* |
| ct693 *pgk* qPCR R | ATAGTTGTGGCACCCGATATAC | Reverse qPCR primer | For qPCR of *pgk* |

| **gBlock Name** | **Sequence** | **Features** | **Usage** |
| --- | --- | --- | --- |
| *pgm* crRNA | tgtgaaagtgggtcttaagacgtcggtactgcatgtgacgcacgtagatcatgca*TTCACCGGTGGAGACGGTTTTCTTATAATGACACC*TAATTTCTACTCTTGTAGAT**GTAATCATCCTTAAATCTTGC**CAAATAAAACGAAAGGCTCAGTCGAAAGACTGGGCCTTTCGTTTTATcaacagcggtctactgaatctgagctagtgcgtgatataattaaaattatattca | Lower case for plasmid overlap and spacer, *italicized* for P_dnaKmut_ promoter sequence, underlined for crRNA scaffold, **bold** for *pgm* targeting sequence, Upper case for rrnB1 terminator | For CRISPRi knockdown of *pgm* |
| *pgk* crRNA | tgtgaaagtgggtcttaagacgtcggtactgcatgtgacgcacgtagatcatgca*TTCACCGGTGGAGACGGTTTTCTTATAATGACACC*TAATTTCTACTCTTGTAGAT**GGTTAAGGATCAGATAAGCAT**CAAATAAAACGAAAGGCTCAGTCGAAAGACTGGGCCTTTCGTTTTATcaacagcggtctactgaatctgagctagtgcgtgatataattaaaattatattca | Lower case for plasmid overlap and spacer, *italicized* for P_dnaKmut_ promoter sequence, underlined for crRNA scaffold, **bold** for *pgk* targeting sequence, Upper case for rrnB1 terminator | For CRISPRi knockdown of *pgk* |
